# Hydrogen Sulfide Biology and Its Role in Cancer

**DOI:** 10.3390/molecules27113389

**Published:** 2022-05-25

**Authors:** Saadullah Khattak, Mohd Ahmar Rauf, Nazeer Hussain Khan, Qian-Qian Zhang, Hao-Jie Chen, Pir Muhammad, Mohammad Azam Ansari, Mohammad N. Alomary, Muhammad Jahangir, Chun-Yang Zhang, Xin-Ying Ji, Dong-Dong Wu

**Affiliations:** 1 Henan International Joint Laboratory for Nuclear Protein Regulation, School of Basic Medical Sciences, Henan University, Kaifeng 475004, China; saadullah@henu.edu.cn (S.K.); kakakhan3514@gmail.com (N.H.K.); 13323950805@163.com (Q.-Q.Z.); chj104753201139@henu.edu.cn (H.-J.C.); 2 Department of Surgery, Miller School of Medicine, University of Miami, Miami, FL 33136, USA; mxr2481@med.miami.edu; 3 Henan-Macquarie University Joint Centre for Biomedical Innovation, School of Life Sciences, Henan University, Kaifeng 475004, China; pir@henu.edu.cn; 4 Department of Epidemic Disease Research, Institute for Research & Medical Consultations (IRMC), Imam Abdulrahman Bin Faisal University, P.O. Box 1982, Dammam 31441, Saudi Arabia; maansari@iau.edu.sa; 5 National Centre for Biotechnology, King Abdulaziz City for Science and Technology (KACST), P.O. Box 6086, Riyadh 11442, Saudi Arabia; malomary@kacst.edu.sa; 6 Department of Psychiatric and Mental Health, Central South University, Changsha 410078, China; jahangir.masoom@csu.edu.cn; 7 Department of Thoracic Surgery, The First Affiliated Hospital of Zhengzhou University, Zhengzhou 450052, China; 8 Department of General Thoracic Surgery, Hami Central Hospital, Hami 839000, China; 9 Kaifeng Key Laboratory of Infection and Biological Safety, School of Basic Medical Sciences, Henan University, Kaifeng 475004, China; 10 School of Stomatology, Henan University, Kaifeng 475004, China

**Keywords:** endogenous gases, hydrogen sulfide, signaling pathways, cancer, translational medicine

## Abstract

Hydrogen sulfide (H_2_S) is an endogenous biologically active gas produced in mammalian tissues. It plays a very critical role in many pathophysiological processes in the body. It can be endogenously produced through many enzymes analogous to the cysteine family, while the exogenous source may involve inorganic sulfide salts. H_2_S has recently been well investigated with regard to the onset of various carcinogenic diseases such as lung, breast, ovaries, colon cancer, and neurodegenerative disorders. H_2_S is considered an oncogenic gas, and a potential therapeutic target for treating and diagnosing cancers, due to its role in mediating the development of tumorigenesis. Here in this review, an in-detail up-to-date explanation of the potential role of H_2_S in different malignancies has been reported. The study summarizes the synthesis of H_2_S, its roles, signaling routes, expressions, and H_2_S release in various malignancies. Considering the critical importance of this active biological molecule, we believe this review in this esteemed journal will highlight the oncogenic role of H_2_S in the scientific community.

## 1. Introduction

Like nitrogen oxide (NO) and carbon monoxide (CO), hydrogen sulfide (H_2_S) is a biologically active gas found in mammalian tissues. H_2_S plays an essential role in mediating the many molecular, physiological, and pathophysiological functions in living systems [1,2]. H_2_S is considered to be involved in developing and progressing various diseases, ranging from non-malignant [3,4] to carcinogenic [5,6,7].

In its carcinogenic roles, H_2_S is involved in both the inhibition and advancement of cancer [5,8,9,10], while in its non-carcinogenic function, it has been found that H_2_S plays a significant role in the development of oral diseases [11,12], respiratory diseases [13,14], cardiovascular diseases [15,16,17], and common kidney diseases [18,19,20]. Consequently, these molecules have a tremendous impact on, and regulate the function of the mammalian system [1,21]. H_2_S is the third-most abundant naturally occurring gas, after NO and CO [21,22,23,24], and profoundly affects the body’s production and regulation of enzymes.

H_2_S has become widely accepted as a critical signaling molecule in cancer biology due to its unique chemistry, molecular reactivity mechanisms, capacity to change proteins, and active participation in numerous redox processes with metal. H_2_S has been implicated in a variety of physiological processes linked to the cell cycle and tumor progression, including angiogenesis, tumor growth, cellular and mitochondrial biogenesis, tumor blood flow, migration and invasion, metastasis, protein sulfhydration, epithelial-mesenchymal transition, DNA repair, and chemotherapy resistance [25,26,27,28,29,30,31]. There are numerous publications describing the potential roles of H_2_S in cancer [32], such as Cao et al. [32] and Shackelford et al. [27]. Cao et al. explain the synthesis, metabolism, measurement, and modulation of H_2_S as a novel treatment in cancer [32], and Shackelford et al. investigated the role of H_2_S in cancer, with an emphasis on the molecular processes through which H_2_S promotes cancer development, dedifferentiation, metastasis, and proliferation. However, considering the bloom and growth of knowledge on H_2_S biology, an up-to-date and exclusive publication is worth considering.

The present review outlines advancements in the understanding of H_2_S in cancer management by highlighting its functional involvement in critical cellular process such as programmed cell death, DNA repair, ferroptosis, immunomodulatory, and downstream impacts on cellular activities, with its role in signaling and mediating a dual role in cancer. The review also emphasizes the therapeutic potential of H_2_S donors alone or in combination with other therapeutics. Although H_2_S is the hallmark for many processes at the cellular level in mammalian systems, physiological functions are entirely understandable and clarified. However, to better understand its significant role and function in cancer-related processes, the review also describes in-depth knowledge on the synthesis of H_2_S.

## 2. Physiological and Pathological Roles of H_2_S

Previous studies have shown that H_2_S takes part in an extensive range of physiological and pathological situations, such as vascular relaxation, neuronal activity, angiogenesis, glucose metabolism, energy production, atherosclerosis, ischemia-reperfusion (I/R) injury [33,34,35], vasodilatation [36,37], anti-inflammation [38,39], anticancer [40], and cardioprotection [41]. However, there are several arguments on the function of H_2_S in cancer growth and development. In recent years, several reports have recommended that the endogenous or exogenous production of H_2_S may establish two different roles in forming cancer cells [42,43]. 

According to Chiku et al.’s study, human CSE profligacy occurs in various reactions that produce H_2_S from cysteine and homocysteine [44]. In the presence of alpha-ketoglutarate, it can also be generated via platelet-rich plasma (PRP)-independent 3-mercapto-pyruvate sulphate transferase (3-MST) or cysteine aminotransferase (CAT) [45]. In mitochondria, free H_2_S may be oxidized by sulfhydryl reductase (SQR) and may be methylated through sulfhydryl-S-methyl transferase in the cytoplasm [46,47]. Additionally, free H_2_S is emitted by biological liquids when it joins molecules with metal and methemoglobin [48]. H_2_S production increased in vivo in the presence of phosphodiesterase inhibitors, inorganic sulfide salts, and organic H_2_S donors [49]. Sodium hydrosulfide and P-(4-methoxyphenyl)-p-4morpholinodithiophosphoric acid (GYY4137) are two common H_2_S donors [50]; SG-1002 [51], NOSH-aspirin, ACS67 (a mixed compound of latanoprost and H_2_S releasing moiety) [52,53], and L-cysteine are substrates for the endogenous formation of H_2_S [21,54]. Although a lack of confirmation exists on the normal range of H_2_S in altered cells and the events that lead to its variation in tumor cells, existing research proposes that only a small amount of H_2_S is required for maintaining cellular activities, and that any change in its level, whether increasing or decreasing, has a significant impact on cancer-modulating cellular activities.

## 3. Endogenous Production of H_2_S

**Figure 1:**
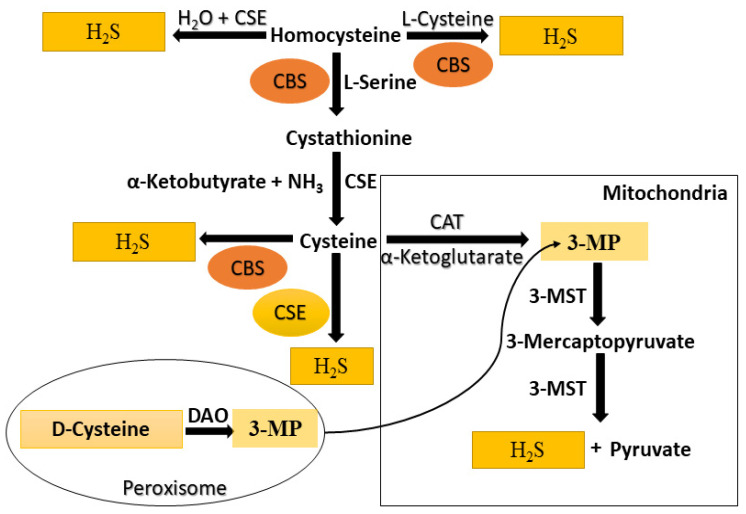
A schematic illustration of the biosynthesis of endogenous H_2_S in mammals. H_2_S, hydrogen sulfide; H_2_O, water; CBS, cystathionine β-synthase; CSE, cystathionine γ-lyase; NH_3_, ammonia; α-ketoglutarate; 3-MST, 3-mercaptopyruvate sulfurtransferase; CAT, cysteine aminotransferase; 3-MP, 3-mercaptopyruvate; DAO, D-amino acid oxidase.

Recent experimental research has shown that all mammals, including humans, produce H_2_S enzymatically [55]. The commonly observed enzymatic pathway includes CSE and CBS, two pyridoxal-5-phosphates (PLP). Further, another enzyme, 3-MST, which is non-PLP-dependent, act in unison with CAT and in the presence of α-ketoglutarate to produce H_2_S from L-cysteine. Both enzymes are co-localized in the cytosol and mitochondria [56,57,58]. Additionally, a study has shown that D-amino acid oxidase can catalyze D-cysteine to form an achiral α-ketoacid, 3-mercaptopyruvate, which is further processed through 3-MST into H_2_S in both brain and kidneys [6] (Figure 1). The produced H_2_S is instantly released or converted to acid-labile sulfur or bound sulfane sulfur and stored in mammalian cells [33,59,60]. The catabolism of H_2_S can occur through mitochondrial oxidation to sulfate and thiosulfate, excretion from the kidneys or lungs, hemoglobin-mediated scavenging, and thiol methyltransferase and rhodanese-mediated methylation to generate methanethiol and dimethyl sulfide [33,61,62]. CBS and CSE are present in the fluid portion of the cytoplasm, and are also called cytosolic enzymes with certain tissue distributions. CBS is mainly present in the central nervous system (CNS) and occurs in the kidney, liver, uterus, pancreatic islets, placenta, and ileum.

In contrast, CSE can be generated in the heart, kidney, uterus, ileum, vascular smooth muscle, and liver. CSE is the mainly applicable H_2_S-producing enzyme in the cardiovascular system [55,63]. 3-MST has been detected in mitochondria and in the cytosol, whereas CBS and CSE are predominantly cytosolic [64]. They have been found in the kidney, liver, heart, lung, brain, thoracic aorta, thymus, and testis, which is very important for H_2_S production in the vasculature brain [55,63].

H_2_S may be directly discharged, released, or accumulated in acid-labile sulfur, or bound during the cells’ enzymatic pathway [63]. Moreover, H_2_S can be endogenously produced via either enzymatic or non-enzymatic pathways [55,63]. The endogenously non-enzymatic production of H_2_S occurs via glucose, organic and inorganic polysulfides present in garlic, and elemental sulfur and glutathione [63].

Non-enzymatically, H_2_S can be generated from glucose by a different process such as glycolysis (>90%) or phosphogluconate through nicotinamide adenine dinucleotide phosphate (NADPH) oxidase (<10%) [55,63]. When glucose interacts with methionine, homocysteine, or cysteine, gaseous sulfur compounds—methanethiol and H_2_S—are formed. The direct reduction in glutathione and elemental sulfur also produces H_2_S. Elemental sulfur is reduced to H_2_S by reducing equivalents of the glucose oxidation pathway such as NADH or NADPH [65,66]. Furthermore, garlic and garlic-derived organic polysulfides might influence H_2_S formation in a thiol-dependent manner; for example, diallyl trisulfide (DATS), diallyl disulfide (DADS), S-allyl cysteine (SAC), and diallyl sulfide (DAS), respectively [35,63,67,68]. Similarly, L-cysteine is produced by two pyridoxal-5′-phosphate-dependent enzymes such as CBS and CSE, and with the joined action of 3-MST and CAT, H_2_S is produced [57,69] (Figure 1).

The biosynthesis of cysteine in mammals is the primary pathway that plays an essential role in producing H_2_S. CSE and CBS are pyridoxals-5′-phosphate-reliant enzymes in the cytosol. Thus, CBS is expressed mainly in the central nervous system [70], catalyzing H_2_S and cystathionine from homocysteine by cysteine declination and degradation [71]. Simultaneously, CSE is most abundantly expressed in the cardiovascular system and produces cysteine, ammonia, and α-ketobutyrate from cystathionine, and H_2_S from cysteine metabolism. The third pathway consists of 3-MST and CAT, which are mainly present in the cytosol and mitochondria of different tissues such as neuroglia cells, kidneys, cardiac, and liver [71]. In the presence of α-ketoglutarate, CAT catalyzes the alteration of cysteine to 3-MST, which can generate H_2_S and pyruvate from 3-MP degradation and breakdown [72].

The production and biogenesis of H_2_S in the human body mainly occurs via two main routes, via particular endogenous enzymes, and as a result or a by-product of microbial metabolic pathways inside the gut microbiota, especially in sulfate-reducing bacteria. Amusingly, it was found from the evaluation of germ-free against conservative mice that intestinal microbes control homeostasis, not just in the gut, but also systemically in different organs and tissues [59]. Specifically, microbiota occurrence has been attached to upper levels of free H_2_S, not just in the colon and cecum (intestinal tracts), but also in plasma. Furthermore, in germ-free mice compared to conventional animals, the traditional animals showed higher levels of bound sulfane sulfur in the fat, lungs, and plasma, with lower cysteine levels and higher CSE activity in most tissue organs [59]. Additionally, persulfides and polysulfides are considered to be secondary sources of H_2_S, which may be produced or generated endogenously, or from nutritional intake [73]. The main three human enzymes studied to produce H_2_S endogenously are CBS, CSE, and 3-MST [45].

## 4. Upregulation of Different H_2_S-Producing Enzymes in Cancer

Different studies have revealed the altered expression and role of these three enzymes in developing several cancer cells, as explained below and summarized in Table 1. Szabo et al. revealed that CBS is highly expressed in colon cancer cells [34]. Later on, several other studies reported on the upregulation of CBS, CSE, and 3-MST in various cancers [74,75,76,77]. The functional role of H_2_S-producing enzymes in cancer has been studied comprehensively, and it has been revealed that cancer cells upregulate the ability of H_2_S-producing enzymes to assist with bioenergetics functions, in order to exploit ATP generation for the purpose of increased growth, migration, and proliferation [34,76,77,78,79,80,81,82,83,84,85].

### 4.1. CBS Expression in Cancer

In 2013, a study comparing human colon cancer specimens with healthy mucosa tissue presented selective up-regulation of CBS in cancer tissue, whereas low CBS expression levels were observed in non-cancerous peri-tumor tissue. The other H_2_S-producing enzymes, CSE and 3-MST, showed no upregulation in tumor cells. With the consequent testing of HCT-116, HT-29, and Lovo (colon adenocarcinoma-derived cell lines), it was observed that CBS is selectively upregulated, in concert with NCM356 (a non-malignant cell line of colonic epithelial cells). Cell fractionation studies were also conducted to investigate CBS location in colon cancer cells, with the results showing that CBS is habitually localized to the cytosol, but mitochondrial translocation is also possible [86,87]. A study using HCT116 cancer cells revealed that CBS is present in cytosol and mitochondria. As per an estimate, homogenates of specimens collected from patient-derived colon tumors and cancer cell lines derived from colon homogenates showed an increase in the production rate of H_2_S. This response was inhibited using the prototypical CBS inhibitor compound amino-oxy-acetic acid (AOAA) [86].

CBS was overexpressed in primary epithelial tissues, ovarian cancer, and multiple ovarian cancer cell line specimens similar to colon cancer cells. After examining 200 primary epithelial ovarian cancer patients’ tissues using microarrays, Bhattacharyya and colleagues found high levels of CBS expression, predominantly in a common histological variant of serous carcinoma. Tumors with higher-grade cancers and serous histology contain higher levels of CBS. Previous studies have shown strong levels of CBS expression in the FIGO stages (I and II) of ovarian cancer [88]. In further experiments using quantitative real-time PCR (RT-PCR) along with immunoblotting, CBS mRNA and protein expression were compared with the control in a variety of cell lines of ovarian cancer, which showed high levels of expression for CBS mRNA and protein in a non-malignant ovarian surface epithelial cell line (OSE) [88].

In melanoma cancer cells that express more CBS than usual, Panza and colleagues identified the various types of congenital nevi (combinational, functional, and dysplastic). According to a study of primitive human melanoma, CBS and 3-MST were also present at a high but variable level. In contrast, different human melanoma cell lines (Sk-Mel-5, Sk-Mel-28, A375, and PES 43) with normal epidermal melanocytes (NHEM) do not exhibit CBS expression [89]. Despite these facts, minimal literature is available to identify significant changes in the expression levels of various H_2_S-producing enzymes for multiple cancers. Guo et al. reported that CBS and CSE could be found in prostatic epithelium normal tissue, while only CSE was found per acinar stroma cells. An androgen-dependent prostate cancer cell line (LNCaP) demonstrated noticeable degrees of CSE and CBS expression.

In contrast, an epithelial cell line of the normal prostatic peripheral zone (RWPE-1) showed low observable expression of both CBS and CSE [90]. Similarly, in many other prostatic cancer cell lines, both CBS and CSE expression and reduced expression of CBS and CSE were observed. Both CBS and CSE are primarily localized to the cytoplasm. Guo and colleagues tested the effect of dihydrotestosterone (DHT) on CBS and CSE expression, and found that DHT initiated an increase in CBS and CSE expression in LNCaP cells [90]. Additional cancer cell types showing increased CBS expression consist majorly of cells in the NC160 collection [91], myeloma, and biliary tract carcinoma [92]; breast cancers consistently showed the highest increase, as did renal tumors [93]. The functional effect of variation in H_2_S synthesis in the cancer cell lines was not investigated.

CBS knockdown of cancer cells inhibited xenograft development and neovessel density, indicating a function for endogenous H_2_S in tumor angiogenesis. Unlike CBS, suppressing CSE (whose expression was intact in colon cancer) did not affect tumor development or bioenergetics. In conclusion, H_2_S produced from CBS serves to (i) maintain colon cancer cellular bioenergetics, thereby supporting tumor growth and proliferation, and (ii) promote angiogenesis and vasorelaxation, consequently providing the tumor with blood and nutrients. CBS-derived H_2_S has been identified as a tumor growth factor and anticancer medication target [34]. CBS detection can also be observed in several other cells. CBS expression is low or absent in the normal prostate peripheral zone epithelial cell line RWPE-1, while CBS expression is high in the androgen-dependent prostate cancer cell line LNCaP [90].

Like non-malignant colon mucosa cells, epithelial cell lines from colon cancer exhibit selective CBS upregulation and increased H_2_S development [36]. The expression of CBS was significantly increased in protein and mRNA levels in ovarian cancer cells [12,94]. In addition, human breast cancer cells MDA-MB-468, MCF-7, and Hs578T showed significantly higher CBS levels than normal breast cells [95]. Recent studies have shown that estrogen-related receptor a1 (ERRa1) activates the transcription factor Sp1 and plays a critical role in controlling CBS expression in different cell types [96]. Further analyses were needed to determine whether ERR1 plays a role in expressing CBS in cancer cells, such as the expression of CBS mRNA in hepatocellular carcinoma (HCC). On the other hand, hypoxia and radiation conditions can dramatically increase the amount of CBS in the human hepatoma cell line, HepG2 [97]. CBS is inhibited in colon and colorectal cancer by promoter methylation, and CBS, mediated by methylation, can be reversed genetically or pharmacologically [98].

CBS expression can also contribute to the development and progression of human glioblastomas [99]. CBS expression was not detected in leukemia cells, indicating that CBS is more prevalent in solid tumors [35,93]. Therefore, the expression of CBS is significantly altered in several human tumor types, and the expression of CBS has a few characteristics. CBS mechanisms must be characterized to determine their specific roles in tumor cell proliferation, invasion, and metastasis.

### 4.2. CBS Function in Cancer

Szabo et al. compared human colon cancers with normal tissue from the patient’s lining. Further, several cell lines derived from the colon’s adenocarcinoma were examined [34], including the selective regulation of CBS, which was contrasted with the non-malignant epithelial cell line [34]. Szabo et al. explored the localization of colon cancer cells using CBS cell fractionation [34]. In addition to its translocation from the cytosol to the mitochondria, CBS is considered a cytosolic enzyme [86,87]. According to Szabo et al. [34], a study on the localization of HCT116 cancer showed that CBS is in the cytosol and the mitochondria. It is predicted that colon cancer patients’ serum homogenates and cell lines produce significantly more H_2_S suppressed by AOAA, a prototypical CBS inhibitor [34]. Szabo et al. examined the role of HBS derived from CBS in colon reproduction, passage, and in vitro invasion. CBS can be genetically silenced or pharmacologically inhibited to prevent the proliferation, migration, and invasion of HCT116 cells [34].

Moreover, S-Adenylyl-L-methionine (SAM), an allosteric CBS activator of this compound, enhances the proliferation of HCT116 cells at low concentrations [34,86]. By combining genetic and pharmacological approaches, AOAA inhibits CBS. The genetic silencing of CBS or the pharmacological inhibition of CBS inhibits the proliferation, migration, and invasion of HCT116 cells [34]. Additionally, S-Adenylyl-L-methionine (SAM), a CBS allosteric activator of this compound, stimulates HCT116 cell proliferation at low concentrations [34,86]. CBS-derived H_2_S proliferative and pro-migratory characteristics are likely suitable for Akt/PI3K signaling stimulation, as early studies have established that H_2_S exogenous donors excite the migration of HCT116 cells via pathways activation [100]. In summary, the H_2_S produced by CBS results from its stimulation of mitochondrial and bioenergetic actions. Additionally, Szabo et al. revealed that silencing CBS and inhibiting AOAA decreased the bioenergetic activity of HCT116, which requires basal electron transport. The respiratory reserve capacity bioenergetic parameter is an increase in mitochondrial oxygen consumption in response to a mitochondrial uncoupling agent [34]. CBS inhibition obscures mitochondrial function and the glycolytic function of HCT116 cells [34], a finding that can be explained by H_2_S’s documented stimulation effect on GAPDH activity [101], a key enzyme in the glycolytic pathway. SAM increased the bioenergetic activity of HCT116 cells at low concentrations, similar to the CBS activator SAM’s allosteric effect on proliferation [86].

Experiments with nude xenografts of HCT116 cells or patient tumor tissue (PDTX) confirmed the results in vivo. A combination of the pharmacological inhibition of CBS with AOAA and CBS silencing significantly reduced the formation of tumor xenografts. Szabo et al. have reported CBS inhibition effects in vivo [34]. In some cases, it may have paracrine effects on the tumor microenvironment. Moreover, the absence of CBS blocked the density and convolution of CD31-positive vessels between the tumor tissues, indicating reduced tumor angiogenesis. In addition, AOAA was shown to condense blood flow per tumor and act as a local vasodilator when injected directly into the tumor parenchyma [34]. CBS inhibition with AOAA inhibited the metastatic spread of HCT116 cells from the cecum to the liver, and AOAA oxaliplatin inhibited the metastatic spread in the same model [102]. Primary epithelial ovarian cancer tissues and several ovarian cancer cell lines are over-expressed with CBS. Bhattacharya et al., proposed that primary ovarian tumors expressed high levels of CBS. CBS levels are typically higher in tumors with serous histology and a higher degree of cancer [103].

The expression of CBS is already identifiable in most of the early stages (FIGO Stage I and II) of the ovarian cancers studied [88]. Another study by a group used quantitative RT-PCR and immunoblotting to compare the expression of CBS mRNA and protein levels in different ovarian cancer cell lines with a non-malignant superficial ovarian cell line as a control (OSE) [88]. Bhattacharyya and colleagues investigated the functional role of CBS-derived H_2_S in inhibiting the proliferation, migration, and invasion of ovarian cancer in vitro, using a combination of genetic and pharmacological approaches. The regulation or inhibition of CBS has been shown to inhibit cell proliferation, and treatment with AOAA, particularly at higher concentrations, also reduces cell viability. As Bhattacharyya and colleagues investigated the intracellular mechanisms underlying these acts, they discovered that controlling or inhibiting CBS reduces the essential antioxidant glutathione’s (GSH + GSSG) intracellular content and causes apoptotic cascades. The absorption of the intracellular antioxidant may cause this latter effect after CBS inhibition/suppression. Another inevitable result of CBS cessation or inhibition is an increase in reactive oxygen species in the cells; this may be secondary to antioxidant depletion or correlated with mitochondrial function changes. Finally, CBS styling impacts on intracellular signaling in A2780 cells: Bhattacharyya and colleagues discovered that CBS suppression increased the expression of p53 while reducing the expression of the NF-kB RelA/p65 subunit [88].

H_2_S increases mitochondrial function and cell bioenergy in ovarian cancer cells, as seen in colon cancer cells. According to Bhattacharya and colleagues, CBS reduced oxygen consumption in mitochondria, and similar effects were observed when ovarian cancer cell lines were treated with a CBS, AOAA inhibitor. In addition to CBS suppression, CBS inhibition has been shown to increase mitochondrial ROS production, decrease NAD/NADH ratios, decrease ATP synthesis, and increase ADP/ATP ratios [88]. The results of these experiments were confirmed in nude mice transplanted with A2780/CP-20 xenografts. There was a significant reduction in tumor weight (approximately 40%) and a consistent, more pronounced (approximately 70%) decline in tumor nodules with CBS. Based on Ki-67 staining, CBS silencing decreased cancer cell proliferation. As well as inhibiting the angiogenesis surrounding the tumor, CBS silencing inhibited angiogenesis (as in the colon cancer study mentioned above) [88]. Bhattacharyya and colleagues demonstrated that CBS inhibition sensitizes cancer cells to chemotherapy, both in vitro and in vivo. CBS silencing reduced cisplatin’s IC50 rate in A2780 cells by more than half. Using cisplatin alone, 80–90% of patients felt distressing symptoms after being treated with cisplatin independently. CBS siRNA and cisplatin, on the other hand, significantly reduced tumor weight and nodules [88].

### 4.3. CSE Expression in Cancer

CSE has been shown to play a critical role in various types of cancer cells, as indicated by a study inhibiting CSE by shRNA, or a study inhibiting DL propargyl glycine and cancer cell proliferation and invasion in the human colon SW480 [103]. Human colon cancer HCT116 cells express mRNA and protein levels of CSE [34,104]. H_2_S/CSE is also involved in hepatocytes, as they correlate with H_2_S output and are critical for cell proliferation [105]. CSE’s expression and functional activity have also been determined in C6 glioma cells [106]. A CSE expression analysis revealed that PC-3 prostate carcinoma cells are the primary source of endogenous H_2_S [107]. Molecular mechanisms of CSE may be crucial for cancer development and progression, and this requires further investigation. Cancer prevention and treatment can be enhanced by identifying and producing specific CSE inhibitors [35].

The expression of CSE and CBS is also reduced in prostatic cancer cell lines [107]. The cytoplasm is the primary location of CBS and CSE. A study conducted by Guo and colleagues found that DHT increased the expression of CBS and CSE in LNCaP cells [90]. Drugs targeting CSE or CSE inhibition do not affect the proliferation, migration, or growth of HCT116 cells [34]. Additionally, it was found in SW480 that CSE expression is high in the cells. The levels were further enhanced by activating the Wnt pathway in the cells. Pharmacological or genetic inhibition of CSE impairs cell proliferation in vitro, and blocks CSE with propargylglycine (PAG). Using SW480 cells inhibited by CSE, tumor growth in mice bearing tumors is slowed down [103]. CBS and other CSE cell lines develop H_2_S in larger amounts than other colon cancer cell lines, which is consistent with the conclusion that H_2_S promotes cell proliferation.

### 4.4. CSE Function in Cancer

Researchers have indicated that CSE may be the main H_2_S-producing enzyme in peripheral tissues, based on their studies of mice lacking CSE [108]. CSE has different evidence-based arguments to support its involvement in diverse cancers. Researchers have investigated CSEs in prostate cancer by studying LNCaP cells [90], late-onset in PC-3, and LNcaP-B [109]. The over-expression of CSE with NAHS stimulates the proliferation of prostate cancer cells [109]. This suggests a role for CSE/H_2_S pathways in prostate cancer. There have been reports of CSE-induced outcomes in melanoma [110] and gastric cancer [40,111], and H_2_S donors foremost to cell apoptosis in both types of cancer. However, it is still unclear whether CSE enhances or prevents cancer. A recent study did not explain what CSE inhibition or removal means in these cells. Studies have shown that CSE helps cells to survive in hepatocellular carcinomas [105,112]. Yin et al. have found that the PI3K/AKT pathways control CSE in QGY-7703 and SMMC-7721 hepatoma cell lines [112]. In HepG2 cells, the knockdown of CSE by siRNA significantly inhibits cell proliferation, as found in another study [105]. Several vital pathways are involved in this type of cancer, including the Wnt/B-catenin pathways [103]. It also suggested that siRNA knockdown or the pharmacological inhibition of CSE has pro-cancer effects on colon cancer. This is supported by evidence from experiments with SW480 and HCT116 [103].

NaHS stimulated cancer cell proliferation by activating extracellular signal-regulated kinase pathways in HCT116 and SW480 [104]. In recent research, the expression level of CSE correlates positively with urothelial cell carcinoma of the bladder [113]. However, the exact contribution of CSE remnants to bladder cancer development needs to be explored.

### 4.5. Expression of 3-MST in Cancer

3-MST is expressed by all somatic cells. The expression of 3-MST by tumor cells is not unexpected. Human neoplastic cell lines have also been reported to express and activate 3-MST, with activities that are higher than CSE. Therefore, it is presumed that 3-MST is the primary source of H_2_S, rather than CSE [114,115]. Additionally, 3-MST expression and catalytic activity have been reported in hepatoma [116,117], glioblastoma and astrocytoma [115], colon cancer [10,34,118], renal carcinoma [119], urothelial cancer [113,120], lungs adenocarcinoma [121], and melanoma cell lines [89] with marginal concentrations. Cancer cell lines with stem-like properties and multidrug resistance express 3-MST induced by stress or cytotoxic stimuli [117,118]. Despite this observation, 3-MST may not offer any cytoprotective or valuable benefits in advanced or drug-resistant cancer. 3-MST expression is also evident in primary tumors, including human gliomas, with higher malignant grades [122]. Accordingly, glioblastoma containing ipsilateral hemispheres produced higher amounts of H_2_S than controls [123]. A relatively low proportion of 3-MST was expressed in human melanoma (25–50%), but a significant proportion of the subunit E was not available for analysis [89]. The expression of 3-MST in resections of bladder cancer [113,120], colon cancer [10], oral squamous cell carcinoma [124], lung carcinoma [125], and adenoid cystic carcinoma [126] was significantly higher than in healthy tissues.

### 4.6. The Function of 3-MST in Cancer

In 1960, Kun’s group reported an initial investigation using the metabolic reaction of 3-mercaptopyruvate in tumors and healthy cells. During their investigations, the author claimed that the 3-MST system in cancer cells appears to have reduced enzymatic activity than normal tissues [127]. Later on, Wlodek et al. observed similar observations of 3-MST activity and cysteine aminotransferase activity [128]. However, this perception was changed because the tumor cells displayed an increased oxidative stress burden. 3-MST might be inactivated in the oxidized state, while an ex-vivo enzymatic assay cannot duplicate living cells’ conditions. Noticeable differences in the results of these new papers may also have occurred because the 3-MST activity of the cancer cells was compared with homogenates of liver and kidney; amongst all of the parenchymal organs, the liver and kidney have the highest expression of 3-MST. Even if the 3-MST activity was lower than the actual level, there was still substantial activity to confer major functional roles by focusing on the 3-mercaptopyruvate functional effects and the effects of 3-MST silencing Hepa1c1c7 (murine hepatoma cell line). The 3-MST substrate, in lower concentrations, induced a bio-energetic stimulatory impact, whereas an inhibitory effect was higher. With the silencing of 3-MST, it has been observed that the cell’s basal bio-energetic function was slight, nonetheless, it was considerably suppressed; the 3-mercaptopyruvate stimulatory effect was undetectable [116]. After 3-MST silencing, the mitochondria of the isolated cells, in an estimation for their ability to generate H_2_S in reaction from 3-mercaptopyruvate, were prominently suppressed [116]. The stimulatory bio-energetic effects facilitated by 3-mercaptopyruvate are associated with directing electron donation to the electron transport system in mitochondria, because SQR silencing of an enzyme obligatory for this electron donation reduced the 3-mercaptopyruvate bio-energetic stimulatory effect [116]. An additional study to check the 3-MST silencing functional effect used a lung adenocarcinoma cell line (A549). After 3-MST silencing, a reduction in the cells’ proliferation rate was observed, suppressing mitochondrial DNA repair frequency [125].

Instead of reducing 3-MST, silencing enhanced the bio-energetic function of these cells. Due to the effects of 3-MST silencing, the bio-energetic variations may change in a specific direction. There may also be differences in cell types [129]. Interestingly, renal cell carcinoma (RCC4) silencing of 3-MST did not alter its effects [119]. Recently, it has been found that 3-MST inhibitors play a predominant role in many health conditions, including cancers [74]. The 3-MST system plays a role in the microenvironment of tumor cells. The production of 3-MST-derived H_2_S by endothelial cells has been shown to occur during cell proliferation, migration, vascular relaxation, and angiogenesis, specifically under hypoxic conditions [130]. However, bioenergetics and metabolism play a critical role in regulating endothelial cells with the 3-MST system [130].

Furthermore, experiments will be required to determine the value of these findings and the function of the tumor microenvironment. For a better understanding of tumor growth and angiogenesis, more information is needed on the possible role of 3-MST in in vivo studies [131]. Recently, its effects on proliferation, migration, and bioenergetics have been confirmed in murine colon cancer cells [81]. According to the published literature, the role of the 3-MST system extends beyond producing H_2_S. Studies with 3-MST inhibitors and 3MT silencing should be anticipated in terms of its effects on the redox processes that 3-MST regulates, in combination with the H_2_S effects that 3-MST induces. Simulating 3-MST would impair interactions between 3-MST-mediated proteins, possibly resulting in functional consequences. 3-MST is physically associated with enzymes such as L-cysteine de-sulfurase NFS1 and the rhodanese-like protein MOCS3 [132].

## 5. Dual Role of H_2_S in Cancer

**Figure 2:**
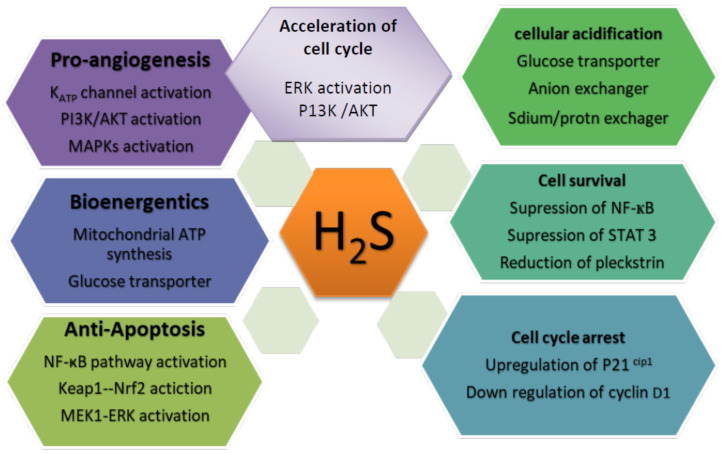
Dual role of H_2_S in cancer.

In cancer, the function of H_2_S depends on the donor’s supplementation, cancer types, and concentration. Figure 2 illustrates how these donors promote and inhibit cancer, as shown here.

### 5.1. The Cancer-Promoting Effect of H_2_S

Mammal cells are currently envisaged to respond to H_2_S as the bioenergetics stimulator at low concentrations. According to Goubern et al., sulfides are have a high affinity to mitochondria in mammals, making them a suitable energetic substrate, even at low concentrations [133]. Thus, the mitochondrial enzyme SQOR is an electron donor that self-regulates parallel to coenzyme Q, in addition to complexes I and II [116] for various cellular bioenergetic functions. However, 3MP can enhance the process [116]. Consequently, H_2_S-mediated mitochondrial respiration can only aid cancer development in the presence of sufficient oxygenation. Additionally, H_2_S, as a substrate for mitochondrial respiration, increases intramitochondrial cAMP levels and induces the persulfidation of mitochondrial ATP synthase and lactate dehydrogenase [116,118]. Glibenclamide inhibits p38 phosphorylation and the migration of endothelial cells after exposure to H_2_S [134], which indicates that p38 is involved in the proangiogenic process induced by H_2_S. H_2_S mediates persulfidation by stimulating the KATP channel and influencing downstream effects, as demonstrated by Mustafa et al. [135]. H_2_ promotes ischemia-induced angiogenesis in several experiments by upregulating HIF-1a expression [136]. The hypothesis that H_2_S contributes to hypoxia-induced angiogenesis during cancer development is highly plausible, although insufficient evidence supports it. The pro-angiogenic activity of H_2_S was first discovered in the late 2000s. H_2_S from NaHS, when used experimentally, promotes the proliferation of endothelial cells, migration, and tubular formation [134]. NaHS also stimulated blood vessel growth and branching when exposed to chicken chorioallantoic membranes. The proangiogenic effects of H_2_S have also been established in rat models [134]. The inhibition of CSE by pharmacological agents or genetic deletion restricted VEGF-induced angiogenesis [134], implicates H_2_S as a physiological angiogenic agent. The PI3K/AKT pathway, mitogen-activated protein kinase (MAPK), and ATP-sensitive potassium (KATP) channels have all been shown to mediate the proangiogenic effects of H_2_S [72,134]. H_2_S has also been shown to promote angiogenesis in endothelial tumors. Using a well-established model of tumor angiogenesis, Puppo et al. discovered that NaHS enhances the migration of endothelial cells isolated from breast carcinomas (B-TECs). In the absence of VEGF, the inhibition of CSE inhibits the migration of B-TECs, suggesting H_2_S is a crucial contributor to both exogenous and endogenous breast cancer angiogenesis. In rat models of colon cancer [34] and ovarian cancer [88], CBS silencing inhibited tumor growth and neovessel density. H_2_S can promote tumor growth by promoting angiogenesis and by transporting nutrients and oxygen to cancer cells. However, in cancer biology, it is essential to note that high concentrations or doses of NaHS may also suppress angiogenesis [137]. This indicates that the pro-angiogenic movement may occur only in low or endogenous H_2_S concentrations. H_2_S has an anti-apoptotic effect, including a defensive effect against apoptotic stimuli [138,139]. Additionally, anti-apoptotic activity has been reported in several cancer cells, including colon cancer [140], hepatocellular carcinoma [141], and neuroblastoma [142]. Additional research has shown possible underlying mechanisms, including the activation of the nuclear factor kappa-light-chain enhancer in activated B cells [143], the activation of the keap1 transcription factor NF-E2-related nuclear factor 2 (Nrf2) [144], and the activation of the MEK1-ERK pathway [140], mediated by H_2_S-linked persulfidation.

The cell cycle is a series of highly well-defined events that control the transition from cellular quiescence (GO) to proliferation, and attest to the high loyalty of the genomic transcript. In eukaryotes, the cell cycle is divided into four phases: gap phase 1 (G1), DNA synthesis phase (S phase), and gap phase 2 (G2), in which the cell prepares for division and the mitosis phase (M phase). Distinct chromosome separation and cell division occur during the M process [145]. The breakdown of normal cell cycle regulation is a common feature of human cancer [146,147]. A recent study has shown that NaHS can function as a proliferative factor by increasing PKB/AKT and ERK expression in oral squamous carcinoma [94]. Although the treatment of HCT 116 cells with NaHS for 24 h effectively and significantly reduced the GO–G1 population and increased the S-phase cell population, treatment with H_2_S effectively and significantly reduced the expression of p21 proteins, which are considered CDK inhibitory proteins [104]. Similarly, the downregulation of CSE disrupts the G1/GO process and reduces cell number in the S phase while increasing cell population in the S phase in the human hepatoma cells BEL-704 [112]. These studies concluded that H_2_S acts as a proliferative factor in cancer by encouraging cell cycle progression.

Cancer cells can proliferate indefinitely by evading arrest in the cell cycle. Recent evidence shows that H_2_S can accelerate or prolong the cell cycle in diverse cell types, including cardiomyocytes, cancer cells, and endothelial cells [107,148]. Here, exogenous H_2_S inhibits the expression of regulatory genes in the cell cycle and increases proliferating nuclear antigens and cyclin-dependent kinase 4 [94]. H_2_S was investigated for its acceleration effect on the cell cycle in colon cancer [104] and hepatoma cells [105]. This fundamental signaling mechanism may be linked to the activation of the ERK and AKT pathways [94,104,105], as the inhibition of ERK or AKT phosphorylation has been shown to inhibit the cell cycle significantly, thereby accelerating the effect of H_2_S on squamous cell carcinomas and colon cancer cell lines [94,104]. Although not explicitly reported, the persulfidation of MEK1 demonstrates the fundamental mechanisms of H_2_S-induced ERK activation. However, the molecular mechanisms of the critical role of AKT in the development of human cancer are still unclear [149]. An explanation and clarification of this would have a significant effect. 

### 5.2. Anti-Cancer Effect of H_2_S

**Figure 3:**
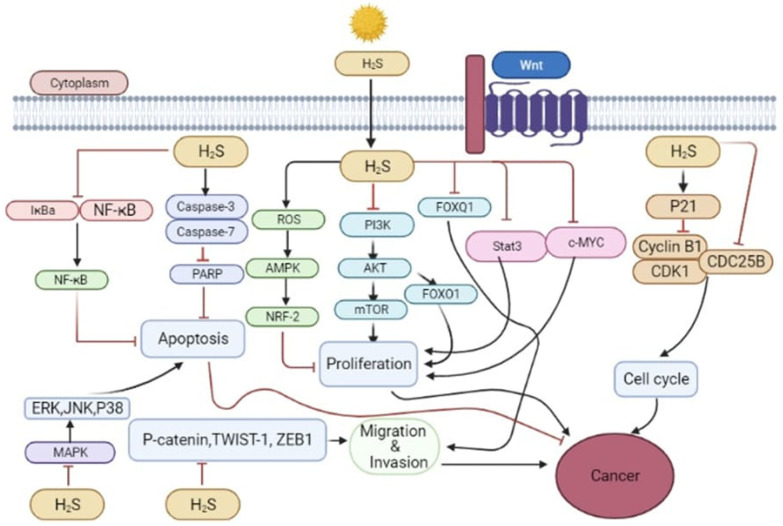
Schematic diagram of pathways of H_2_S and H_2_S donors and their derivatives on cancer. H_2_S and H_2_S donors participate in regulating several pathways to induce apoptosis and proliferation.

The prolonged exposure of cancer cells to high H_2_S concentrations will lead to their death, while normal fibroblasts are unaffected. Figure 3 illustrates the possible mechanisms underlying the antagonization of cancer growth by H_2_S. H_2_S regulation determines the normal functioning of the cells, and depending on the cell type, any dysregulation (upregulation and downregulation) in endogenous H_2_S levels is associated with the development and metastasis of cancer [150,151,152]. Researchers have found that H_2_S-synthesizing enzymes are increased in various human malignancies, including colon cancer, prostate cancer, breast cancer, urothelial, ovarian, oral squamous cells, and thyroid cancers, and a worse prognosis mediates the tumor to advance stages [124,153,154]. To reveal these associations between H_2_S levels and cancer progression, many novel types of research have recently been published in reputed journals [155,156,157,158,159,160]. Recently, two novel studies from China, published in a cancer letter, unknotted this highly recommended and awaited work, and evaluated the potential impact of H_2_S inhibition and H_2_S donation, respectively, on cancer cells [5,56]. Exposure studies have indicated that 5-(4-hydroxyphenyl)-3H-1, 2-dithiol-3-thione (ADT-OH) is a commonly used H_2_S donor for breast cancer cells. HA-ADT suppresses the growth of human breast cancer cells by inhibiting the PI3K/AKT/mTOR and RAS/RAF/MEK/ERK signaling pathways [56].

Similarly, promising results were achieved by inhibiting the CBS, a significant contributor to H_2_S synthesis out of three essential enzymes; its expression heals cancer by reversing acquired resistance to 5-FU in colon cancer cell lines [5]. These two studies are of utmost importance for paving insights into the physiological workings of H_2_S in cancer cells and providing a baseline with implications on the prospect of developing cancer therapies by targeting H_2_S levels in the human body. In adherence to the results of increased expression of Bax and Bcl-2, which mediate apoptosis-related cancer cell death and the destruction of signaling pathways upon the exposure of H_2_S donors achieved in [56], in recent experiments, it has been evaluated that co-treatment with DATS (Dially thiosulfate, a H_2_S donor) and Dex (dexamethasone) significantly inhibits sphere formation, colony formation, and the proliferation of multiple myeloma cells by inducing apoptosis and cell cycle arrest. In addition, treatment mediated an increase in the expression of miR-127-3p and inhibited PI3K, p-AKT, and p-mTOR pathways [161]. In another aspect of the H_2_S role against cancer, a novel study published on the pharmacological inhibition of H_2_S-producing enzymes, showed that the induction of significant changes in gene and protein expression has the potential to pharmacologically induce the mesenchymal-to-epithelia transition (MET) and disturb the EMT/MET balance in colon cancer, which evaluates and designates H_2_S as a potential contributor in anti-metastatic mechanisms against cancer [85].While according to recent study, I194496, a new CSE inhibitor, suppresses human TNBC development and metastasis by downregulating numerous signalling pathways [162]. In follow-up investigations on [49], it has been further elucidated that ADT-OH prevents IκBɑ breakdown, leading to decreased NF-B activation and subsequent downregulation of the anti-apoptotic proteins XIAP and Bcl-2. More crucially, it prevents FADD from being degraded by ubiquitin by suppressing the production of MKRN1, a FADD E3 ubiquitin ligase [53].

The metabolic process of glycolysis is found within cancer cells [163], and it is designed to increase glucose production and convert lactate into energy. Lactate accumulation can cause inflammation and stress in the cells. An acidic microenvironment may enhance angiogenesis and metastases in cancer cells derived from intracellular acid synthesis [164]. Therefore, it is a promising strategy for treating cancer to target intracellular pH regulators [165]. GYY4137 (200 to 1000 nM) increased cancer cell glycolysis via cumulative glucose uptake. It temporarily inhibits the export of intracellular acids by blocking the function of anion exchangers (AE) and sodium/proton exchangers (NHS) [166]. H_2_S catabolism to H_2_SO_4_ must not be abandoned entirely, as it can also lead to subsequent intracellular acidification. Consequently, uncontrollable intracellular acidification occurs in a panel of cancer cell lines, leading to cell death [166]. GYY4137 did not show such an effect when tested with ZYJ1122, a sulfur-free control compound [8,166], which means that H_2_S alone was responsible for its behavior.

In mouse models, GYY4137 has significant antitumor activity. In their study, researchers studied the impact of GYY 4137 on non-cancerous fibroblast cells such as Wi-38 and MCF10A [8,166]. They discovered that GYY4137 did not cause intracellular acidification. By stimulating the activity of the Cl¯/HCO3¯ transporter, NaHS (10 µM–1 mM), in contrast, reduces the intracellular pH of vascular muscle cells [167]. It has been demonstrated that the same findings were replicated in primary cultured glial cells but not in SH-SY5Y neuroblastoma cells [168].

Cell cycle dysregulation proved to be involved in cancer progression [169]. Thus, cell cycle arrest induction is an effective way to treat cancer cells. Several studies have reported the H_2_S suppressive effect on the cell cycle switch. Sproargyl-cysteine (SPRC) acts as an H_2_S donor, causing G1/S phase cell cycle arrest and subsequent apoptosis in vitro and in vivo in the gastric cancer cell line SGC-7901 [40]. In a group of colon cancer cell lines (HT-29, SW116, and HCT116), NaHS (0.4 to 1 mM) induces cell cycle arrest at G1/S, likely by upregulating the cyclin-dependent kinase inhibitor p21Cip1 [170]. Furthermore, the inductive effect of GYY4137 on cell cycle arrest in many cancer types has been suggested [8,171]. For example, Lu et al. discovered that GYY4137 inhibited the transition of the G1/S cell cycle by downregulating cyclin D1, thus inhibiting tumor growth in the subcutaneous HepG2 xenograft model [171]. GYY4137 induced a partial arrest of G2/M in a breast cancer cell line (MCF7), but the underlying mechanism is unknown [8]. H_2_S induces cell cycle arrest in cancer cells, since neither NaHS nor GYY4137 induces cell cycle arrest in normal fibroblast cells in the above studies [8,170].

Dysregulation of the cell cycle has been shown to play a crucial role in cancer progression [169]. Therefore, the inhibition of cell cycle arrest is beneficial in cancer treatment [147]. Although many research studies have shown that the H_2_S suppressive effect is crucial for cell cycle transition, H_2_S donors and Sproargyl-cysteine both cause cell cycle arrest at the G1/S step and subsequent apoptosis in the gastric cancer cell line SGC-7901, in vitro and in vivo [40]. NaHS-induced cell cycle arrest at G1/S in HCT116, SW116, and HT-29 colon cancer lines may be due to the upregulation of the cyclin-dependent kinase inhibitor p21Cip1 [105]. However, GYY4137 was also used to investigate the effects of the cell cycle on various cancer types [8,171]. Lu et al. demonstrated that GYY4137 inhibited the transition of the G1/S cell cycle through downregulation of cyclin D1, suppressing tumor development in the subcutaneous HepG2 xenograft model [171]. Additionally, it has been shown that GYY4137 causes a partial arrest of G2/M in breast cancer, but the primary mechanism has not been identified [8]. To our delight, H_2_S tends to precisely arrest the cell cycle in cancer cells, as NaHS or GYY4137 do in normal fibroblast cells [8,170]. However, the molecular mechanisms by which H_2_S causes those effects remain unknown. In eukaryotes, the cell cycle is divided primarily into three phases: G1 to S, G2 to M, and M to G1 [172]. The precise transition from the G1 to the S phase is crucial for regulating cell proliferation, and failure to do so can lead to oncogenesis [172]. SPRC treatment of SGC-7901 gastric cancer cells for 24 h will significantly inhibit proliferation and migration by blocking the cell cycle in the G1/S phase [173]. The administration of GYY4137 for 24 h inhibits the cyclin D1, inhibiting the transition of the G1/S cell cycle and tumor growth in the Xenograft model of the subcutaneous HepG2 [171].

Several studies have found that NaHS can arrest the cell cycle and promote the expression of the p21Cip1 protein in colon cells treated for 12 to 24 h [170]. According to recent research, the G2/M checkpoint may be a potential target for anticancer drugs [174]. The treatment of breast cancer cells with 400 mM GYY4137 for 5 to 8 days results in the G2/M cell cycle arrest, accompanied by an increase in the G1 cell population. Consequently, H_2_S appears to inhibit the proliferative activity of cancer cells by specifically blocking the cell cycle and protecting non-cancer cells from death [175]. There could be other mechanisms responsible for H_2_S’s anticancer activity. For example, H_2_S can increase E-cadherin levels, which have anti-metastatic effects [176], and inhibit histone deacetylase, resulting in the epigenetic reactivation of tumor suppressor genes [177]. The molecular targets responsible for the pleiotropic effects of H_2_S on biological processes remain unknown, because H_2_S is responsible for a plethora of biological processes. Cancer cells disturb the balance between apoptosis and survival by activating pro-survival pathways in persistently growing cancer cells [178]. NF-κB, a signaling pathway, has been implicated in the development of several cancers, including non-small cell lung cancer, breast cancer, and prostate cancer. In addition to activating NF-κB by persulfidating the p65 subunit, H_2_S has also been shown to inhibit its activation by TNF and lipopolysaccharide [179,180]. Thus, it is not surprising that chronic exposure to H_2_S [89] or donation hybrids [181] causes detrimental effects, including NF-κB inhibition and apoptosis.

In contrast, the molecular mechanism by which H_2_S inhibits NF-κB activity is not well understood, and further research is needed to better understand the mechanism of H_2_S anticancer action. For instance, GYY4137 induces apoptosis in hepatocellular carcinoma cell lines by inhibiting STAT3 activators and downregulating B cell lymphoma 2 through STAT3 [171]. Additionally, chronic H_2_S exposure causes apoptosis in oral cancer cells, probably due to the downregulation of pleckstrin homology-like Domain-A1, an apoptotic suppressor found in this type of cancer [182]. The importance of identifying and discussing the H_2_S-target proteins involved in cell survival pathways must be addressed in the future.

## 6. H_2_S Production and Programmed Cell Death

**Figure 4:**
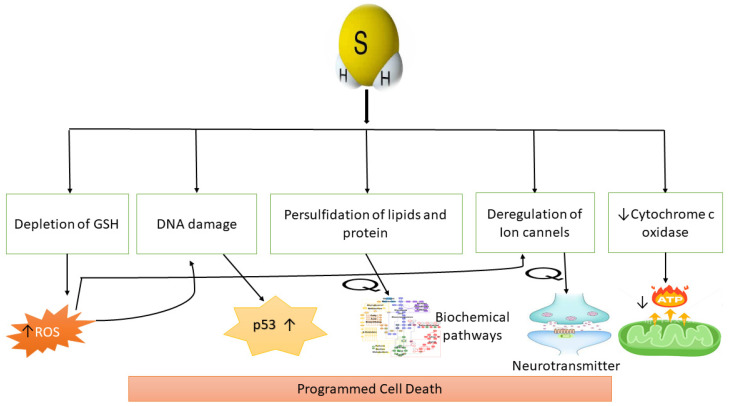
Proposed H_2_S-induced cytotoxicity pathways in mitochondria. H_2_S inhibits cytochrome c oxidase, resulting in reduced ATP generation. H_2_S also impairs calcium homeostasis, resulting in elevated intracellular calcium levels. Reduced glutathione depletion results in reactive oxygen species (ROS). H_2_S causes DNA damage, protein and lipid persulfidation, and ion channel dysregulation, exacerbated by high intracellular ROS levels. These H_2_S-induced actions, taken together, may result in programmed cell death. ↑: Increase or Generation; ↓: Decreased or Reduced.

Programmed cell death (so-called apoptosis) plays a fundamental role in controlling oncogene initiation, unrestrained proliferation, and chemotherapy. Recently, H_2_S production and programmed cell death have been well-investigated in many studies. H_2_S prevents apoptosis in colon cancer cells induced by β-phenyl ethyl isothiocyanate [167]. 6-hydroxydopamine management also contributes to apoptosis in a human neuroblastoma cell line (SH-SY5Y), while NaHS treatment and CBS over-expression decreased cell death [110]. It has also been shown that the H_2_S signaling pathway is crucial to maintaining the proliferation of hepatoma cells. While the inhibition of these pathways prevents these cells from developing, this may be due to mitochondrial apoptosis. However, treatment with NaHS increases cell viability in the hepatic cells, PLC/PRF/5 [111]. Additionally, NaHS treatment alleviates mitochondrial oxidative stress and restores the protective effect of NaHS against mitochondrial toxicity (Figure 4) [112]. Although H_2_S is necessary for increasing the apoptotic ratio of cancer cells (CA9-22), apoptotic markers in normal keratinocytes are unknown [113]. H_2_S sulfhydrates the NF-κB p65 subunit, facilitating its attachment to the co-activator ribosomal protein S3. The anti-apoptotic capabilities of NF-κB are drastically diminished in CSE mutant mice. H_2_S that is released via CSE improves DNA binding and NF-κB gene activation, both being abolished in CSE-deficient animals. H_2_S sulfhydrates the NF-κB p65 subunit, facilitating its attachment to the co-activator ribosomal protein S3. The anti-apoptotic capabilities of NF-κB are drastically diminished in CSE mutant mice [114,122]. Due to these studies, H_2_S appears to mediate anti-apoptosis in the progression of diseases that are associated with extreme cell development and division, including cancer. However, the exact mechanism is unknown.

## 7. H_2_S as a Signaling Molecule and Role in Signaling Pathways

H_2_S acts as a signal molecule in various structures and tissues, including the circulation, nervous system, and organs [117,118]. Endothelial cells, smooth muscle cells, mitochondria, endoplasmic reticulum, and transcription factors play a role in using H_2_S in inflammatory cells [104,183]. Hong et al. demonstrated that H_2_S promotes the proliferation and migration of SW480 cells derived from human colon cancer in vitro, and can contribute to SIRT1 upregulation. CBS increased H_2_S synthesis in ovarian and colorectal cancers, which are critical for the bioenergetics, proliferation, and migration of cancer cells [119]. By activating NF-κB signals, H_2_S increases the expression of IL-6 and IL-8 in periodontal fibroblasts, which can contribute to the stimulation and production of periodontitis [120].

TNF functions as an activator of NF-κB pathways, resulting in increased CSE expression and H_2_S generation. CSE enhanced p65 DNA binding and downstream gene expression in mice lacking CSE. NF-anti-apoptotic B’s activity is dramatically diminished in mice lacking CSE [88]. As a result of this finding, H_2_S has been proposed as an endogenous mediator of inflammation via its increase in the activity of the NF-κB pathway [121]. Similarly, the inhibition of CSE can moderate melanoma cells’ proliferation by inhibiting NF-κB pathways [122]. However, it is worth noting that exogenous H_2_S can suppress the activation of the NF-κB pathway in inflammatory conditions [46,47,123,124]. In particular, Yang et al. suggested that H_2_S acts as an endogenous stimulator for the Keap1pNrf2 pathways [125]. Activating this pathway makes it possible to cause the production of oxidants such as ferritin, S-transferases, and epoxy hydrolase, leading to chronic oxidative stress as the disease develops [126,184]. Zhao et al. proposed that H_2_S assists with DNA repair by triggering the MEK1/REK/PARP1 pathway [127].

By inhibiting the signaling pathway PI3K/AKT/mTOR, treatment with 10^−3^ M NaHS, a donor of H_2_S for 24 h, inhibits the migration, proliferation, and division of human hepatocellular carcinoma cells, inducing cell autophagy [185]. A recent study showed that 24 h treatment with 30 µM NaHS induces autophagy and regulates matrix metabolism in high-glucose mouse glomerular endothelial cells through the LKB1/STAD/MO25 signaling pathway [186]. Numerous studies suggest that H_2_S activates the AMPK-activated protein kinase (AMPK) in rat glomerular epithelial cells, BV2 mouse microglial cells, and C_2_C1_2_ mouse skeletal muscle cells via calmodulin kinase beta (CamKK) [129,130]. This process may act as a checkpoint for signaling pathways such as PI3K/SGK1/GSK3 and PI3K/AKT [132]. Excessive autophagy may contribute to the vascular endothelial dysfunction associated with diabetes when induced by severe oxidative stress. For example, 12 weeks of therapy with 100 mol/kg NaHS (i.v. or i.p. injection) could protect the mouse’s arterial endothelial cells from oxidative stress by blocking the Nrf2-ROS-AMPK signaling cascade [133]. The findings indicate a novel therapeutic strategy for diabetes-induced endothelial damage to the arterial wall [133]. According to a recent report, intragastric administration of the NaHS solution at a dose of 8 mol/kg/day for four months can minimize the death of smoking-induced autophagy cells in rats by modulating the AMPK/mTOR signaling pathway [169]. NaHS (0.2 mg/kg injected over 10 s, followed by a 2 mg/kg/h infusion) can provide biochemical myocardial defense in cardioplegia and cardiopulmonary bypass by activating ERK/1 and attenuating caspase-independent apoptosis and autophagy [187].

## 8. Protein Sulfhydration and Cancer 

Protein sulfhydration is a post-translational protein alteration in which a sulfur atom is added to a reactive protein cysteine, resulting in a -SSH or a persulfide group being created. Cysteine persulfide is formed when an oxidized cysteine derivative combines with a sulfide or sulfide oxidation product. Protein sulfhydration predominantly inhibits, with most activating events being driven by the persulfidation-induced inhibition of a negative regulator [75]. This section will look at a few cancer-related self-hydrated proteins that have received much attention.

Similarly, polysulfides are also considered to be the key players in mediating the different oncogenesis pathways. A recent study indicates that a high CBS and CSE expression level indicates a poor prognosis [188,189]. A study of 186 stage III or IV ovarian tumors using surface-enhanced Raman spectroscopy discovered elevated CSE expression associated with cisplatin resistance, a poor prognosis, and higher tumor polysulfides. Moreover, enhanced polysulfide production boosted cisplatin resistance in ovarian carcinoma cell lines with high CSE expression. CSE suppression improved ovarian tumor cell susceptibility to cisplatin, which was caused by increasing the phosphorylation of histone H2AX and reducing polysulfides. In vitro, hydrogen polysulfides reduced cisplatin-induced DNA damage, with minor damage being seen as the number of sulfur atoms in every polysulfide increased [188]. Polysulfides inactivate PTEN, a tumor suppressor gene product, by adding sulfate sulfur to the cysteine’s active site, decreasing PTEN phosphatase activity [190]. This, and many other polysulfide-related procedures, is likely to result in a thankless function in cancer.

## 9. H_2_S-Mediated Persulfidation of NF-kB 

NF-kB is a dimeric transcription factor family that is triggered through a wide range of stimuli and is involved in immunological responses, inflammation, and cancer [191]. A modified biotin switch assay revealed that NaHS administration increased cell invasion and NF-kB p65 cysteine 39 sulfhydration in the prostate cancer PC3 cell line. Maximum p65 cysteine 39 sulfhydration was seen at NaHS concentrations ranging from 10 nM to 10 M, indicating that sulfhydration occurred at physiologically relevant donor dosages [191]. In contrast, CSE knockdown inhibited sulfhydration and cell invasion. PC3 cells harboring the p65C38S mutant had decreased NaHS-induced invasive ability, but no sulfhydration. These occurrences were avoided by re-expressing wild-type p65 expression. In a murine xenograft animal model, the p65C38S mutant had fewer metastases than the wild-type p65. These data imply that p65 subunit Cys sulfhydration is vital in prostate cancer spread [192].

The NF-kB pathway inhibits apoptosis by increasing the production of anti-apoptotic proteins such as TNFR-associated factor (TRAF)-1, TRAF-2, caspase-8-c-FLP, and cellular inhibitors of apoptosis [193], signifying that H_2_S can operate as an endogenous activator of the Keap/Nrf2 pathway. By stimulating the Keap1-Nrf2 pathway, H_2_S can stimulate the expression of enzymes such as glutathione S transferases, epoxide hydrolase, and ferritin, allowing cancer cells to adapt to prolonged oxidative stress and progress [194,195]. Even so, the mechanism by which the persulfidation of Keap1 leads to the liberation of Nrf2 is still indistinguishable, and more research is required. By stimulating the MEK1-ERK- PARP1 pathway, H_2_S can aid in DNA repair, as in Zhao et al. [109]. H_2_S persulfates MEK1, in particular, in cysteine-341, and thereby, it influences downstream effects. Many cell types are affected by H_2_S, including cardiomyocytes, cancer cells, and endothelial cells [48,196]. For example, exogenous H_2_S (NaHS, 200 to 500 lM) lowers the expression of cell cycle control genes, such as replication protein A70 and retinoblastoma protein 1. However, in certain oral cancer cell lines, it increases the production of proliferating nuclear antigen and cyclin-dependent kinase 4, which leads to cell proliferation [94]. Studies have found that H_2_S can speed up the cell cycle in colon cancer cells [104] and hepatoma cells [105]. Cell lines from squamous cell carcinoma and colon cancer [94,104,105], ERK or AKT phosphorylation partially inhibit the H_2_S-induced acceleration of the cell cycle [94,104]. Although not explicitly confirmed, it has been suggested the persulfidation of MEK1 may cause H_2_S-induced ERK activation. The molecular mechanism by which H_2_S phosphorylates AKT is, however, unknown. In light of AKT’s crucial role in creating human cancer [149], deciphering this will be significant.

## 10. H_2_S and DNA Repair

ATR kinase suppression reduced cellular H_2_S levels, indicating a function for H_2_S in DNA repair. Low cellular H_2_S levels enhanced ATR kinase activity, as determined through CHK1 phosphorylation; high amounts of H_2_S, on the other hand, prevented ATR ser-435 phosphorylation, which is a hallmark of ATR kinase activity [31,197]. ATRCHK1 pathway activation is increased in various tumor types [198,199]. Furthermore, elevated ATR protein, phospho-ATR, and phospho-CHK1 expression are associated with a poor prognosis in bladder, ovarian, and breast cancers [199,200,201]. Because the ATR kinase controls H_2_S concentrations, increases in ATR and CHK1 may increase H_2_S production. Furthermore, targeted ATR inhibition is being investigated in cancer treatment [202]. This cancer treatment method might perhaps partially suppress H_2_S production [203]. These potential cancer-related occurrences should be investigated.

## 11. H_2_S and Immunomodulation in Cancer

H_2_S has complex and robust immune system-regulating effects in normal and pathologic conditions, with decreased functioning being frequently observed at low and high H_2_S concentrations [204]. Considerable amounts of data suggest that H_2_S-induced immune modulation has a role in cancer [95]. CBS, as previously indicated, is found at the cancer cell membrane in breast cancer, where CBS-derived H_2_S protects the cancer cells from activated macrophage-generated ROS [95]. Melanoma-bearing mice were also injected with a vehicle or a vehicle + DATS, and melanoma development, splenic myeloid-derived suppressor cells (MDSCs), dendritic cells, and T cells were assessed [150]. MDSCs contribute to cancer development by inhibiting tumor-specific T cells. DATS injection suppressed melanoma development and decreased the number of MDSCs in the spleen, blood, and tumor microenvironment, while boosting CD8 T-cells and dendritic cells. DATS administration also dramatically reduced the MDSCs’ immuno-suppressive activity, restoring T cell function and T cell-mediated tumor growth suppression, suggesting that H_2_S donation controls tumor development via immune system regulation [103,150]. These findings suggest that H_2_S can stimulate and prevent tumor development by modulating the immune system.

## 12. H_2_S and Ferroptosis

Cell death is crucial for mammalian growth and homeostasis, and it is thoroughly interwoven with an organism’s physiological function and pathological condition [205]. Cell death orchestration, either geographically or temporally, is crucial for the growth of numerous human illnesses [206]. Cell death may be classified into four forms for most of the cells in the body: apoptosis, necrosis, autophagy, and pyroptosis [207]. A novel non-apoptotic cell death mechanism mediated through an iron-dependent lipid peroxidation damage was called “ferroptosis” in 2012 [208]. Ferroptosis is a type of cell death that is caused by cell membrane damage, via glutathione peroxidase (GPX) activity failure and intracellular lipid peroxide, which is accompanied by iron-dependent reactive oxygen species production (ROS) [208,209]. Its physiology, genetics, and biochemical properties are distinct from apoptosis, necrosis, autophagy, and pyroptosis [210].

Ferroptosis manifests in cells primarily as decreased mitochondrial volume, increased bilayer membrane density, and the reduction or disappearance of mitochondrial cristae, with no nuclear concentration or chromatin marginalization [211]. In general, the mitochondrion regulates ROS generation, ferroptosis, and the cell cycle, and it has been linked to a variety of cancers, including lung cancer. Furthermore, irradiation and hypoxia stimulate the activity of mitochondrial stress pathways to survive in a harsh environment. Compared to normal cells, tumor cells consume more ROS and iron due to their increased metabolic rate [212,213]. As a result, the changes above inhibit ferroptosis in tumor cells. Many studies on ferroptosis and cancer are currently being conducted. CSE generates H_2_S endogenously, acting as a cardiovascular protective enzyme and as the key enzyme for l-cysteine (a precursor of GSH) [214]. H_2_S has an antioxidative effect by increasing GSH content and reducing ROS [215]. In addition, to reduce ferroptosis, GPX4 activity is inhibited while the Xc system is kept stable [216,217]. GSH depletion is an essential feature of ferroptosis. The homocysteine/methionine cycle produces GSH, an intracellular antioxidant [218]. L-cysteine, a precursor of GSH, is also a significant generator of H_2_S. Growing amounts of data suggest that H_2_S increases GSH synthesis to reduce oxidative damage. In a neurocyte, mitochondrial H_2_S synthesis raises the amount of GSH and encourages its redistribution to the mitochondria, therefore protecting the neurocyte from oxidative stress [219]. H_2_S increases GSH production in a myotube to ameliorate impaired glucose homeostasis [220]. H_2_S donor NaHS treatment boosts GSH synthesis, reducing oxidative stress and postponing cell senescence [144]. According to findings, exogenous (NaHS injection), H_2_S generation considerably recovered GSH loss in response to HHP. As a result, H_2_S downregulation-mediated GSH reduction might constitute a unique mechanism of ferroptosis in HHP [221]. The beneficial effects of H_2_S on ferroptosis suppression have recently been demonstrated in research. H_2_S inhibits ferroptosis by suppressing ALOX12 acetylation and controlling the stability of the xCT (the functional submit of the Xc system), according to Wang and Chen et al. [216,222]. Therapy with the H_2_S donor GYY4137 reduces ferroptosis, which helps to reduce acute lung damage [223]. In hepatocellular carcinoma, inhibiting H_2_S generation with the CBS inhibitor CH004 supplement worsens ferroptosis [224]. These investigations show that H_2_S might inhibit ferroptosis, and so provide protection. The study found that administering the H_2_S donor NaHS boosted GPX4 expression while decreasing ROS generation and lipid peroxidation, correcting high hydrostatic-induced ferroptosis.

Furthermore, NaHS reversed RLS3-induced ferroptosis. Altogether, H_2_S reduces ferroptosis, which helps to reduce HHP-induced VSMC dysfunction [221]. Zinc oxide nanospheres (VZnO) can effectively reduce H_2_S content in colorectal cancer, thereby inhibiting the growth of CT26 and HCT116 colorectal cancer cells. Furthermore, removing H_2_S from colorectal cancer cells inhibits tumor growth by activating ferroptosis, a non-apoptotic form of cell death. Biosafety-related toxicological and pathological analyses demonstrated the low toxicity and high safety of VZnO in the treatment of colorectal cancer [225]. Traditional treatment programs employ ferroptosis inducers, and new adjuvants effectively treat lung cancer. As a result, inducing ferroptosis in lung cancer cells has emerged as a novel anti-cancer treatment strategy [226,227]. Ferroptosis, overall, performs a significant role in the development and treatment of cancer.

## 13. H_2_S-Donating Compounds

The cytoprotective properties of H_2_S are being increasingly recognized. Recent studies have identified novel gas sources to restore physiological function to diseased cells or organs. To date, many individuals have been identified as donors. H_2_S compounds occur naturally in garlic [67,228], sulforaphane, and iberin [229,230]. Cysteine-activated H_2_S donors [231] are cysteine analogs [40,180,232] such as S-propyl cysteine, S-allyl cysteine (SAC), S-propargyl cysteine (S-SPRC) and N-acetyl cysteine (NAC), H_2_S-releasing NSAID derivatives [233], and GYY4137 [8,179,234,235]. H_2_S donors, including ADT-OH, NaHS, thiobenzamide, DADS, and DATS, have recently been used for indefinite endogenous H_2_S development [236,237,238,239]. ATB-346 and GIC-1001, two H_2_S donor-based therapies, are being tested in phase II clinical trials [240,241]. Furthermore, aside from promoting coronary and chronic diseases [242,243,244,245,246,247], endogenous H_2_S promotes angiogenesis, accelerates the cell cycle, prevents apoptosis, and promotes the expression of oncogenes separately [34,248]. Tumor growth is inhibited by promoting intracellular acidification, PTEN/Akt, PI3K/Akt/mTOR, and NF-κB pathways, with no discernible adverse effects on animal health [76,100,181,249,250,251]. According to research, isothiocyanates (ITCs) in cruciferous vegetables have been linked to a lower cancer risk or cancer incidence [252]. ITCs are a defensive strategy against infections by glucosinolate hydrolysis triggered by the myrosinase family of enzymes [253]. ITCs that have received the greatest attention include allyl isothiocyanate (AITC), benzyl isothiocyanate (BITC), phenethyl isothiocyanate (PEITC), and sulforaphane (SFN) [254]. AITC (5, 10, 15, and 20 M) induced oxidative stress, as well as the ERK signaling pathway in a human breast carcinoma cell model, which contributed to apoptosis activation (e.g., the upregulation of caspases 3 and 9) and the growth arrest of cells in the G2/M phase (e.g., the increased expression of p21 and the suppression of cyclin B and CDK1), mitochondrial depolarization, and mitochondria-associated protein dysregulation (e.g., reduced Bcl-2 expression and elevated cytochrome c and Apaf1) [255]. In keeping with these findings, Wu et al. (2011) showed that higher ROS levels occur in osteogenic sarcoma cells treated with BITC (7.5 M) and PEITC (10 M) that resulted in an increase in nitric oxide (NO) generation, the dysregulation of mitochondria potential, cell cycle suppression, and death [256]. Exposure to BITC (2.5–20 M) resulted in a substantial reduction in cell viability caused by ROS generation, mitochondrial malfunction, the dysregulation of pro- and antiapoptotic genes, and the activation of multiple caspases in a breast cancer cell model comprising MDAMB231 and MCF7 cells [257]. Furthermore, PEITC (0.5–5 M) was an efficient inhibitor of oral squamous carcinoma cell growth by cell cycle arrest and mitochondrial-dependent apoptosis caused by ROS generation and Ca^2+^ buildup [258]. AITC (1–40 M), on the other hand, inhibited the survival of human A549 and H1299 non-small cell lung cancer (NSCLC) cells in a dose-dependent manner by generating replication stress and sensitizing tumor cells to radiation [259]. Furthermore, it has been demonstrated that even modest doses of PEITC (0.1–10 M) can inhibit cell growth and proliferation in prostate cancer (LNCaP) cells [260]. Finally, the treatment of human colon cancer cell lines with SFN and PEITC (0.1–100 M) resulted in a dose-dependent decrease in proliferation and apoptotic induction [261].

PHI (5–40 M) inhibited cell cycle development in human leukemia cells by altering chromatin histones’ acetylated and methylation states [262]. In comparison, SFN (15 M) treatment produced a decrease in HDAC3 and six activity levels while increasing p21 expression levels in human embryonic kidney 293 cells and human colorectal cancer (HCT116) cells, indicating that SFN might operate as an efficient tumor suppressor agent [263,264]. SFN (15 M) was also an efficient HDAC inhibitor in BPH1, LnCaP, and PC3 prostate epithelial cells, producing growth arrest and apoptosis activation [265].

Furthermore, GSTP1 methylation is crucial in tumor initiation in prostate cancer. In this context, PEITC (0.5–20 M) has been shown to reduce the deacetylation and methylation of the GSTP1 gene, hence reducing the oncogenic process [266]. PEITC (5 M and 7.5 M) and SFN (20 M and 30 M) significantly inhibited the phosphorylation of IKK/IB kinases and p65, as well as NFB subunit nuclear translocation, thereby suppressing the expression of NFB-related genes (e.g., VEGF, cyclin D1, and B-cell lymphoma extra-large (BclXL), causing angiogenesis. Alternatively, it has been observed that the signal transducer and activator of transcription 3 (STAT3) factor are overexpressed in several cancers, encouraging tumor formation and progression [267]. Boreddy et al. (2011) discovered that BITC (5–20 M) decreased the phosphorylation of STAT3 in pancreatic cancer cell lines, which was followed by a reduction in VEGF and MMP2 production, hence inhibiting angiogenesis [268]. Furthermore, it has been postulated that ITCs defend against tumorigenesis by increasing the ubiquitination of oncogenes, therefore favoring their destruction by the proteasome. Both BITC and PEITC have been shown to target USP9x (ubiquitin specific peptidase 9 X-linked), a member of the deubiquitinating enzymes (DUB), promoting the degradation of the antiapoptotic protein Mcl1 (myeloid cell leukaemia1) and the oncogenic fusion protein BcrAbl in various tumorigenic cell lines [269]. Lastly, tubulin, which is recognized to impair microtubule polymerization and, as a result, induce mitotic arrest and death, is another target of ITCs’ antiproliferative impact [270,271,272]. As a result, it is clear that numerous studies confirm the different impacts of ITCs in many malignancies, including ovarian [273], glioma [274], bladder [275], breast [276,277], myeloma [278], prostate [279,280], and colon [281].

BITC induces G2/M phase arrest and apoptosis in human melanoma A375.S2 cells through ROS and multiple mitochondrial and death receptor-mediated signaling pathways [282], and also via the in vitro inhibition of murine melanoma B16F10 cell motility and invasion [283]. NF-κB sensitives colorectal cancer cells to BITC-induced antiproliferation [284]. Recent work shows that STAT3 is an Sp-regulated gene in pancreatic cancer cells that can be targeted by BITC and other ROS inducers, establishing a potential therapeutic strategy for targeting STAT3 [285]. BITC causes apoptosis through increasing ROS, altering Ca^2+^ concentrations, and decreasing mitochondrial membrane potential. A few of these mechanisms have been noticed in glioblastoma GBM8401 cells [286,287], cisplatin-resistant oral cancer CAR cells [287], gefitinib-resistant lung cancer NCI-H460/G cells [288], estrogen-responsive (MCF-7) and estrogen-independent (MDA-MB-231) human breast cancer cells [289], murine WEHI-3 leukemia cells [290], and human melanoma A375.S2 cells. ROS generation caused mitochondrial malfunction by disrupting mitochondrial membrane integrity and causing oxidative damage, which led to apoptosis [291]. Furthermore, BITC has been shown to increase the expression of the pro-apoptotic proteins Bax and Bad while decreasing the expression of anti-apoptotic proteins Bcl-2 and Bcl-xL in breast cancer cells. Furthermore, BITC modulates mitochondrial dynamics in both estrogen-responsive (MCF-7) and estrogen-independent (MDA-MB-231) human breast cancer cells by modulating the proteins involved in mitochondrial fusion–fission [292]. In vivo, the BITC-mediated downregulation of proteins involved in mitochondrial dynamics regulation was discovered in the mammary tumors of MMTV-neu mice fed a 3 mol BITC/kg diet, and BITC oral administration increased the expression of pro-apoptotic proteins caspase-3 and Bax development in GBM 8401 tumor-bearing nude mice [293]. BITC exposure resulted in a considerable increase in ERK phosphorylation in human breast MCF-7 cells [294]. BITC was discovered to inhibit proliferation, induce apoptosis, and halt the cell cycle in U87MG cells. Furthermore, it inhibited SOD and GSH expression and produced oxidative stress in tumor cells. As a result, it is thought that BITC can stop the development of U87MG cells outside of the body [295]. BITC could limit HCC cell growth and cause cell cycle G2/M phase arrest by downregulating the level of cyclin B1, CDK1, and Cdc25c, and upregulating the expression of Weel; AFP was an antagonist in BITC-mediated cell cycle arrest in HCC cells [289]. In bladder cancer cells, BITC stimulates miR-99a production via an ERK/AP-1-dependent mechanism [296], while in another study, Moringin produced from the myrosinase hydrolysis of GMG displayed anticancer effectiveness in human malignant astrocytoma cells [297]. BITC suppresses human oral cancer cells by inhibiting the redox stress–DNA damage response [298]. In vitro, benzyl isothiocyanate inhibits murine WEHI-3 leukemia cells and enhances phagocytosis in BALB/c mice [290].

Therefore, it is essential that antitumor therapies are developed that use H_2_S donors that are less likely to cause side effects [299]. The role of H_2_S donors in cancer is described in Table 2.

## 14. The Potential of H_2_S in Cancer Therapy in Comparison to Other Complex Compounds

Cancer is one of the most significant threats to human existence, and vast sums of money have been committed to its treatment. Traditionally, the discovery of cytotoxic compounds has resulted in the development of anti-cancer drugs. Over several decades, these drugs, linked to alkylating agents and nitrogen mustard, have been beneficial against various cancers. However, they have substantial side effects, since they cannot distinguish between cancer cells and normal cells.

Advances in molecular biology and genomics have revealed the genetic basis of cancer and potential new targets. As a result, the anticancer drug development paradigm has shifted toward molecularly targeted therapy [319]. The introduction of molecularly targeted medicines such as imatinib, gefitinib, and bortezomib demonstrates the paradigm’s efficacy. However, several limitations have emerged in recent years, including (i) cancer cells that can develop resistance to these drugs; (ii) the treatment can be lost if the target changes; (iii) drugs may be challenging to develop for some targets; (iv) due to the heterogeneity of tumor populations, one drug can hardly abolish tumor growth; and (v) the drug may be unable to penetrate solid tumors adequately. This is represented in the emergence of a changing paradigm. Several targets are covered via pharmaceutical cocktails or multiple-targeted treatments, especially for complex disorders like cancer, diabetes, and acquired immune deficiency syndrome [320,321]. Since its licensure, Regorafenib’s effectiveness and safety have been investigated in various clinical trials and real-world studies, giving a wealth of experience and significant insights into its optimal usage in clinical practice. It is critical to understand that the survival benefit of regorafenib is achieved through disease control rather than through tumor shrinkage, and through the proactive management of adverse events, dose optimization, and patient treatments. At the same time, they are critical for patients who are still undergoing therapies [322,323]. Previously, a patent review on efficient complete synthesis methodologies for pazopanib, regorafenib, and lenvatinib as innovative anti-angiogenesis receptor tyrosine kinase inhibitors for cancer therapy has been published by Shiri et al. [324]. Previously, regorafenib dosage management has been reactive. However, the benefits of proactive first-cycle dose optimization have lately been evident, such as the ReDOS method [325]. HFSR is one of the most prevalent side effects linked with TKIs, including regorafenib [326,327,328]. A published meta-analysis of regorafenib studies found a clinically meaningful difference in all-grade regorafenib-related HFSR incidence across tumor types, with more excellent rates in patients with GIST (60%) vs. HCC (50%) and mCRC (47%). Numerous earlier publications have thoroughly discussed well-established guidelines for preventing and managing HFSR (including therapy and dosage changes) [329,330]. Importantly, regorafenib-related HFSR typically develops during Cycles 1–2 and is thus addressed proactively with dosage adjustments rather than therapy termination [322,331]. In CORRECT and CONCUR, dose modifications were used in 67% and 71% of regorafenib-treated patients to manage all AEs, including HFSR. Yet, the overall rate of discontinuation in CORRECT and CONCUR was relatively low (17% and 14%, respectively), with just 1% and 1% of patients ultimately quitting regorafenib following HFSR [322,332]. Post hoc exploratory analyses of the CORRECT and RESOURCE trials show that patients with treatment-related HFSR received more regorafenib benefits than those who did not; notably, a significant OS benefit was observed when HFSR occurred during the first treatment cycle, supporting continued treatment with dose adjustments [333,334]. Similar results have been observed for regorafenib in the REBECCA real-world trial [335], the Japanese mCRC post-marketing monitoring study [336], and the TKIs sorafenib and sunitinib in HCC and renal cell carcinoma [328,337]. Early HFSR after sorafenib therapy in HCC has recently been linked to enhanced treatment response [338]. However, so that these occurrences are identified prospectively, this technique does not influence the preliminary choice of patients most likely to benefit from regorafenib. The search for baseline prognostic biomarkers is continuing.

Numerous studies have been performed to investigate the effects of pazopanib, which also include hematological, hepatotoxicity, gastrointestinal, cardiovascular, metabolic illnesses, and endocrine and dermatological disorders [339,340]. In clinical investing options, pazopanib has been connected to the development of grade 3 or 4 toxicities [341,342]. According to one meta-analysis, there was a 1.4% incidence of fatal adverse events with pazopanib (FAE). Ischemic stroke, impaired liver function, and rectal bleeding had a relative risk of 4.52 [343]. Pazopanib has been linked to hypertension, myocardial infarction, chest discomfort, ischemia, and transient ischemic attack [344]. Lin et al. (2013) found that pazopanib significantly enhanced the chance of hypertension development in cancer patients. In a phase I study of pazopanib patients with advanced cancer, hypertension was the most common adverse effect, affecting 29% of participants [345]. According to a meta-analysis and another study published in 2012, pazopanib can raise the chance of developing hypertension by 40% [346]. According to one report, up to 52% of participants in a phase 2 trial of breast cancer patients receiving pazopanib suffered hypertension [347]. HTN was seen in 40% of patients with advanced RCC treated with pazopanib in a more extensive randomized, double-blind phase 3 study. MI or ischemia occurred in 3% of participants in this same research. Similarly, in a phase 2 study of pazopanib for recurrent glioblastoma, the incidence of HTN was reported to be 37% [348]. In cancer patients, the risk of all-grade hypertension with pazopanib was comparable to that of axitinib [349]. According to Ghatalia et al., pazopanib has a lower incidence of extended QT intervals [226]. Torsade de Pointes has been observed in less than 2% of individuals treated with pazopanib [350]. According to a meta-analysis and another systematic review, pazopanib has also been connected to venous thromboembolism (VTE).

Furthermore, when compared to controls, the risk of VTE is not statistically significant Min et al., 2013. Hepatotoxicity is yet another severe side effect of pazopanib [351]. Pazopanib treatment increased the considerable risk of severe hepatotoxicity in cancer patients, one of the most prevalent reasons for pazopanib termination [352]. A clinical investigation also showed that the combination medication of pazopanib and simvastatin could cause a rise in ALT, with the incidence of ALT elevation being 7% greater in patients treated with the combination therapy than those treated with pazopanib monotherapy [353]. Acute pancreatitis is an uncommon pazopanib consequence [354]. Proteinuria has also been connected to the use of pazopanib. According to Hurwitz et al.’s phase I trial, 5% of people treated with pazopanib had proteinuria, with 3% having grade 3 or 4 proteinuria. Proteinuria was not observed in the phase II research, which comprised 225 individuals with mRCC.

Proteinuria and grade 3 or 4 proteinuria were found in patients with mRCC who received pazopanib as treatment, with an incidence rate of 9% and 1%, respectively, in a larger population (435 persons) in phase III research [355]. In a community setting, the most common adverse effects of pazopanib were nausea (40%), vomiting (44%), diarrhea (52%), and tiredness (56%) [356,357]. A randomized, phase II study of pazopanib excluded 72% of patients with castrate-sensitive prostate cancer owing to grade 1 or grade 2 toxicities such as diarrhea, fatigue, hypertension, and a rise in ALT and AST levels [358]. However, it is associated with a lower incidence and a relative risk of high-grade and all-grade weariness when compared to sunitinib and sorafenib [359,360]. The use of pazopanib in conjunction with other cytotoxic drugs may result in severe and unbearable side effects. As a result, patients should be closely monitored to avoid toxicity [361]. Because both pazopanib and docetaxel are CYP3A4 substrates, the dosage of pazopanib must be lowered to 400 mg when taken together [362].

Lenvatinib was initially characterized as a multitargeted RTK inhibitor that is capable of inhibiting several kinases at nanomole doses (half-maximal inhibitory concentration, IC50) of 4–100 nM in 2008 [363]. In animal tests, lenvatinib significantly reduced angiogenesis, causing tumors to decrease in a mouse model. A further study in a breast cancer model discovered that targeting vascular endothelial growth factor receptor (VEGFR) 3 during angiogenesis and lymphangiogenesis decreased breast cancer spread to the lymph nodes and lungs [364]. An orthotopic malignant mesothelioma mouse model, which has previously been proven to respond to angiogenesis inhibitors, has also shown efficacy. Lenvatinib extended the lives of mice treated with three mesothelioma cell lines [365] and animals with a sarcoma xenograft [366]. Lenvatinib was eventually developed as an orally administered TKI in the tumor. In healthy volunteers or in patients with solid tumors, lenvatinib is easily absorbed and frequently reaches its peak concentration between 1 and 4 h after oral administration [367]. The absorption followed first and zero-order kinetics unaffected by the higher pH of the stomach. Absorption in patients with solid tumors followed a dose-dependent linear pharmacokinetic pattern, with no drug accumulation after once-daily dosing (maximum concentrations after many doses were the same as those after a single dosage) [368]. Dose modification occurs in people who have toxicities.

The initial phase I study, which included 27 patients [369], was conducted in solid tumors on a two-week, one-week-off regimen. Starting at 0.5 mg b.i.d., the dose was gradually increased to 13, 16, and 20 mg b.i.d. No G3 or four toxicities were seen in individuals taking up to 13 mg b.i.d. during cycle 1. When patients were given greater dosages, dose-limiting toxicities (DLTs) emerged. G3 aspartate aminotransferase/alanine aminotransferase rose in one patient at 16 mg b.i.d., and G3 platelet count dropped in two individuals at 20 mg b.i.d. It was determined that lenvatinib at a dose of up to 13 mg b.i.d. given twice a week for two weeks and once a week for one week would most likely have effects. This trial determined that lenvatinib up to 13 mg b.i.d. in a 2-weeks-on and 1-week-off regimen would have a good toxicity profile.

Another phase I trial with 87 individuals was carried out. In a 28 day cycle, the dosage of lenvatinib was gradually increased from 0.2 mg to 32 mg once a day. Analyses of pharmacokinetics were conducted on days 1, 8, 15, 22, and 28. DLT was identified as G3 proteinuria, with an MTD of 25 mg daily [370]. As a result, the current recommended dose of lenvatinib, when taken alone in patients with solid tumors and retained liver function, is 24 mg daily. It was revealed in a dose-finding study based on population pharmacodynamics and exposure-response analysis in patients with HCC treated with CPA that as body weight declined in individuals with HCC, AUC rose. There was an exposure–response relationship, with higher lenvatinib AUC and lower body weight resulting in faster drug withdrawal or dose reduction. The optimum cutoff values for body weight and lenvatinib AUC to predict the group at high risk for early drug discontinuation or dose reduction were 57.8 kg and 2430 ngh/mL, respectively.

Consequently, for patients with HCC CPA 49, initial doses of 12 mg and 8 mg once daily for persons weighing 60 kg were recommended. A phase Ib study combining lenvatinib and everolimus was conducted to establish the safe dosage for RCC. Starting with 12 mg of lenvatinib once daily (*n* = 7) in a three-plus-three pattern, lenvatinib was increased to 18 mg (*n* = 11) and 24 mg (*n* = 2) in conjunction with 5 mg of everolimus, both given once daily. The MTD was calculated to be 18 mg of lenvatinib and 5 mg of everolimus per day [371]. After years of study, we were able to identify a multitargeted TKI that is active in a variety of solid tumor malignancies. However, it is well recognized that treatment-emergent side effects are common and usually result in dose interruption or therapy discontinuation [372]. Dose interruptions of more than 10% had a worse effect than dose interruptions of less than 10% [373]. A lower dose is being studied to determine whether it can reduce toxicity while maintaining efficacy [374].

Furthermore, biomarkers that predict therapeutic efficacy and toxicity must be researched further so that patients are not put in danger. Finally, lenvatinib appears to be helpful in the treatment of brain cancers [375,376], and its ability to inhibit cancers that have progressed to the brain should be studied further.

H_2_S is a potential therapeutic drug with many biological targets and different properties. Unlike damaging chemotherapeutic drugs, H_2_S has favorable effects in different organs, even at concentrations that are capable of preventing tumor formation, as revealed by the slowly releasing donor GYY4137 [235,319,377,378]. As a result, it is thought that the presence of H_2_S is essential for the maintenance of cellular homeostasis in both standard and malignant cells. This is supported by evidence that H_2_S is essential for modulating redox [379] and thiol homeostasis [380]. As a result, H_2_S modulation may disrupt the cellular equilibrium of cancer as a whole, ultimately leading to death. As a result, H_2_S-based therapy has been effective in cancer types [166,380].

Notably, the differences in endogenous H_2_S levels between cancerous and non-cancerous cells and other factors may allow them to tolerate H_2_S supplementation or inhibition differently. This is reinforced by the therapeutic window demonstrated by the H_2_S-based strategy for cancer treatment [166]. Furthermore, H_2_S is a small lipophilic molecule that may easily pass through all cell membranes and become physiologically active [381]. This might have at least two outcomes: (i) H_2_S may significantly affect the tumor microenvironment, which has been associated with tumor development [382]; (ii) H_2_S might be able to enter solid tumors quickly. In comparison to molecularly focused treatments, H_2_S has been demonstrated to influence many targets in cancer cells, potentially overcoming the limits of the molecularly targeted medications stated above. As a result, H_2_S-based therapy may constitute a novel and distinct technique for cancer treatment, despite its infancy.

## 15. Tumor Markers Associated with H_2_S in Bodily Gas and Fluids

Numerous studies have detected higher levels of H_2_S and associated sulfur compounds in cancer-related controls. Higher H_2_S, for instance, has been found in the headspace vapor of stomach contents in patients with gastroesophageal cancer. Higher H_2_S and methanethiol levels have been discovered in colon and lung cancer patients’ flatulence and exhaled air [383,384,385]. Urine thiosulfate concentrations were 50-fold higher in men with prostate cancer than in men without the condition, indicating that urine thiosulfate may help diagnose prostatic cancer in men with low PSA and negative digital rectal exams. Men with benign prostatic hypertrophy had a 5-fold increase in urine thiosulfate, distinguishing hypertrophy from cancer [386]. The amounts of cystathionine and sarcosine in urine have been linked to prostate cancer [93,387,388]. Cysteine, homocysteine, and cystathionine levels were similarly raised in males with recurrent prostate cancer. Patients with several cancers, including endometrial, esophageal, SCC, prostate, colorectal, and breast cancer, had high plasma homocysteine levels [389]. Ultimately, endogenous H_2_S has been used to detect cancer cells and as a cancer biomarker in mice [203,390]. These findings suggest that H_2_S and similar sulfur compounds may be found in high concentrations in body fluids and gases, and may be helpful in cancer diagnosis. H_2_S and similar sulfur compounds in particular might be utilized in cancer diagnosis by measuring substances such as thiols in the blood and urine to track the efficacy of cancer treatment, induce remission, and identify recurrence.

## 16. Conclusions and Future Directions

As the conclusion of this review, we have stated that CSE, CBS, and 3-MST are three enzymes that play an essential role in producing H_2_S in mammals. These enzymes are over-expressed in all cancer types, and show cancer-related properties. The current understanding of H_2_S research firmly reveals that these enzymes are a key player in regulating the proliferation, migration, and the invasion of cancer cells. In other words, CBS, CSE, and 3-MST may serve as new molecular markers and biomarkers for the diagnosis and treatment of cancer. Considering the double role of H_2_S in cancer, H_2_S donors releasing high levels of H_2_S and other pharmacological designed H_2_S inhibitors are attaining much attention, both in research stations and clinical settings. Being functionally active biomolecules, H_2_S has become the most highly investigated molecular target in cancer biology across the globe. 

Although this review has summarized the potential involvement of H_2_S in important cellular events that directly and indirectly mediate cell fate, there is still much to do before using H_2_S-based anticancer drugs in pre-clinical trials. Firstly, no study has investigated the pathways involved in the beneficial effects of H_2_S donors on cancer, such as NaHS, ADT-OH, DATS, and GYY4137. Secondly, extensive research is needed on the link between the production of endogenous H_2_S through CBS, CSE, and 3-MST, and the activation of cyclin-dependent kinases (CDKs), as we know that CDKs have a vital role in the regulation. Thirdly, most studies are evaluating the function of H_2_S in conjunction with other drugs and messengers, including NO, which moderates many physiological and pathological processes. These messenger molecules offer new perspectives on cancer treatment. Thus, further research is needed to clarify its impact on different cancers.

**Figure 5:**
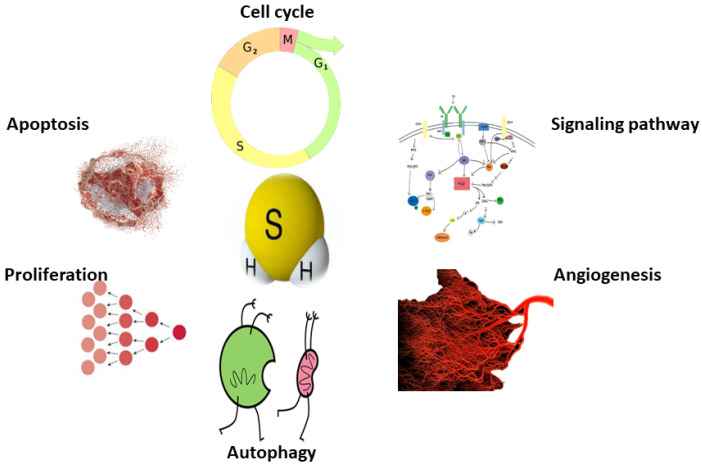
Potential role of H_2_S in function and mechanisms of action in cancer.

Cancer cell signals, survival, and bioenergetics, and perhaps also angiogenesis, depend on the H_2_S system (Figure 5). With the availability of H_2_S pharmacological inhibitors, one might assume these effects translate into functionally detectable in vivo models. Mice bearing tumors could be studied. 

## Figures and Tables

**Table 1:** Change in H_2_S-producing enzymes in different kinds of cancers.

S/No.	Cancer Types	Cell Lines	H_2_S Producing Enzyme		
			CSE	CBS	3-MST
1	Melanoma	A375, WM35, SK-Mel-5, Sk-Mel-28, PES 43	↑	NT	↑
2	Colon cancer	HCT116, HT29	↑	↑	↑
3	Prostate cancer	LNCaP, PC3, LNCaP-B	↑	↑	NT
4	Gastric cancer	SGC-7901	↑	↑	NT
5	Ovarian	OV202, SKOV3, A2780, OVCAR3, OVCAR4, OVCAR5	NC	↑	NT
6	Breast	Hs578T, MCF7, MDA-MB-428	↑	↑	NT
7	Renal	RCC4	↑	↑	↑
8	Thyroid	TPC1, TT, ARO	↑	↑	NC
9	Gliomas	C6, U87MG	NT	NT	↑
10	Hepatocellular Carcinoma	HepG2, PLC/PRF/5	↑	↑	NT
11	Urothelial carcinoma	5637, EJ, UM-UC-3	↑	↑	↑
12	Astrocytoma	U373	NT	NT	↑
13	Neuroblastoma	SH-SY5Y	NT	NT	↑
14	Oral squamous cell carcinoma	OSCC	↑	↑	↑
15	Leukaemia	HL-60, MV4-11	NT	↑	NT
16	Biliary tract carcinoma	EDI-1, TFK-1, HUCCT-1, SNU308, GB-D1, GB-H3	NT	↑	NT

NT: Not tested; ↑: Upregulation; NC: No change.

**Table 2:** Role of H_2_S donors in cancer promotion and inhibition.

S/No	Cancer Types	Cell Lines	H_2_S Donors	Effects on Cancer	References
1	Melanoma	NCI-H929	NaHS	Promotion	[187]
		SKMel 5, SKMel 28 B16F10 A375 S2	GYY4137 BITC	Promotion Inhibition	[89] [282,283]
2	Colon cancer	HCT 116 SW480,	NaHS	Promotion Inhibition	[104] [174]
		HCT 116	GYY4137	Inhibition	[8]
			BITC	Inhibition	[284]
3	Prostate cancer	PC-3	NaHS	Inhibition	[107,109]
		PC-3, Rv1, DU145	BITC	Inhibition	[285]
		LnCaP, DU145	GYY4137	Inhibition	[300]
4	Gastric cancer	SGC 7901	NaHS	Inhibition	[111]
		AGS		Inhibition	[291]
5	Ovarian	A2780, HeyA8, PEA1, PEA2	GYY4137	Inhibition	[300]
		OC	BITC	Inhibition	[301]
6	Breast	MCF-7	NaHS	Inhibition	[8,302]
		MCF-7, MDA-MB-231	GYY4137	Inhibition	[8,300]
		MCF-7, MDA-MB-231	BITC	Inhibition	[303,304]
7	Lung	A549	NaHS	Inhibition	[305,306]
		IMR90, WI-38, A549, H1299	GYY4137	Inhibition	[8,300]
		A549, H661, NCI-H460/G	BITC	Inhibition	[307,308]
8	Thyroid	TPC-1 ARO KTC-1	NaHS NaHS	Promotion Inhibition Inhibition	[309] [310]
		KTC-1	GYY4137	Inhibition	[310]
9	Gliomas	C6	NaHS	Promotion/Inhibition	[311,312]
		U87MG	BITC	Inhibition	[313]
10	Hepatocellular Carcinoma	HepG2, HLE PLC/PRF/5, SMMC-7721	NaHS	Inhibition Promotion	[8,185] [6]
		HepG2	GYY4137	Inhibition	[8]
		Bel 7402,HLE	BITC	Inhibition	[289]
11	Urothelial carcinoma	EJ	NaHS	Promotion	[314]
		DSM cell	GYY4137	Promotion	[315]
		5637,T24	BITC	Promotion	[296]
12	Astrocytoma	U373	NaHS	Inhibition	[244]
		BV2 Cell	GYY4137	Inhibition	[316]
		CCF-STTG1	BITC	Inhibition	[297]
13	Neuroblastoma	SH-SY5Y	NaHS	Inhibition	[244]
14	Oral squamous cell carcinoma	Cal-27, WSU-HN6	NaHS	Promotion	[12,94]
		OG2	BITC	Inhibition	[298]
		SCC9	BITC	Inhibition	[317]
15	Leukemia	MV4-11	NaHS	Inhibition	[244]
		HL-60, MV4-11	GYY4137	Inhibition	[8]
		WEHI-3	BITC	Inhibition	[290]
16	Esophageal carcinoma	EC-109	NaHS	Promotion	[318]

## Data Availability

Not applicable.

## Abbreviations

H_2_S: hydrogen sulfide; NO: nitric oxide; CO: carbon monoxide; CBS: cystathionine-β-synthase; CSE: cystathionine-γ-lyase; 3-MST: 3-mercaptopyruvate sulfurtransferase; CAT: Cysteine aminotransferase; GYY4137: P-(4-methoxyphenyl)-p-4-morpholinodithiophosphoric acid; 4CPI: 4-carboxyphenyl-isothiocyanate acid esters; CNS: central nervous system; CVS: cardiovascular system; GIS: gastrointestinal system; I/R: ischemia-reperfusion injury; SQR: sulfhydryl reductase; DATS: diallyl trisulfide; DADS: diallyl disulfide; SAC: S-allyl cysteine; DAS: diallyl sulfide; ATP: adenosine triphosphate; TNF: tumor necrosis factor; AMPK: AMPK-activated protein kinase; AOAA: amino-oxy-acetic acid; DHT: dihydrotestosterone; HCC: hepatocellular carcinoma; SAM: S-Adenylyl-L-methionine; SPRC: Sproargyl-cysteine; STAT-3: signal transducer and activator of transporter-1; Nrf-2: nuclear factor erythroid-2 related factor; NF-κβ: nuclear factor-kappa B; PI3K: phosphoinositide 3-kinase; ERK: extra cellular signal-regulated kinase; AMPK: AMP-activated protein kinase; TNF-α: tumor necrosis factor-α; TGF-β1: transforming growth factor beta 1; SOD: superoxide dismutase; IL: interleukin; IKK: IκB kinase; Keap1: kelch-like-ECH-associated protein.
